# Assessment of Right Ventricular Adaptability to Pressure Overloading for Critical Therapeutic Decision-Making Processes

**DOI:** 10.3390/jcm15062368

**Published:** 2026-03-20

**Authors:** Michael Dandel

**Affiliations:** German Centre for Heart and Circulatory Research (DZHK), 10785 Berlin, Germany; mdandel@aol.com

**Keywords:** right ventricle, pressure overloading, adaptability to pressure overloading, pulmonary hypertension, right heart failure, echocardiography

## Abstract

Right ventricular pressure overloading [RVPO] with secondary maladaptive RV remodeling and progressive myocardial dysfunction in patients with pulmonary hypertension associated with left-sided heart diseases [PH-LHDs] and in those with pulmonary arterial hypertension [PAH] still remains one of the most complex challenges in cardio-pulmonary medicine. Despite the advances in the optimization of diagnostic tools and the expansion of treatment options, there is still a great need for further research to gain a better understanding of the major pathophysiological mechanisms involved in both the RV responses to PO and to find new possibilities to stop the progression of the alterations inside the pulmonary arterial circulation [PAC]. This article summarizes current knowledge about the particularities of the RV structural and functional responses to abnormal PO and also provides an overview of the benefits and limitations of the currently available tools for clinical evaluations of the RV adaptability to high afterload. A major focus of this review relates to the possibilities for obtaining evidence about the existence of a still remaining adaptability to a normal afterload in an over-burdened RV, in case of abolition of the pathological PO and, in this regard, to also evaluate the clinical usefulness of the RV adaptability estimation for certain critical therapeutic decisions. Among the most important conclusions of this updated overview are: 1. Whereas single parameters are insufficiently reliable for the evaluation of RV dysfunction and for predictions of its prognostic relevance across the whole spectrum of RVPO, properly selected and integrated multiparametric approaches had meanwhile unequivocally proved that they can usually become sufficiently reliable. 2. Multiparametric approaches can substantially improve the prediction of a preserved RV responsiveness to the abolition of its steady PO by reversal of RV maladaptive remodeling and by the normalization of RV pump function. Such a prediction, which can be decisive for therapeutic decision-making especially in candidates for ventricular assist device [LVAD] implantation or thoracic organ transplantation, can have a crucial impact on patient survival. 3. The complex and temporally highly variable interactions between certain structural and functional changes in both the PAC and in the hemodynamic overloaded right-sided heart, as well as between the two ventricles, can often hamper the interpretation of certain changes in the measured parameters and even relevantly alter their reliability. Additionally, the progressive aggravation of a secondary tricuspid regurgitation [TR] has a particularly high negative (often also misleading) impact on the diagnostic and prognostic relevance of RVPO evaluations.

## 1. Introduction

Secondary RV dysfunction is a hallmark of advanced left-sided heart failure [LHF] and severe pulmonary disorders induced by vascular and/or parenchymal alterations, which explains the fact that RVPO can have numerous and very varied causes [1]. Although the most common causes of secondary RV dysfunction are the PH-LHD and the PH associated with lung diseases, also rarer causes like PAH, PH associated with pulmonary artery obstructions (e.g., pulmonary embolism or chronic thromboembolic PH) and pulmonary valve [PV] stenosis can induce massive RVPO associated with RV failure [RVF] [1,2,3,4,5].

Until the early 1980s, the crucial role of the RV in enabling the best possible function of the cardiovascular [CV] system has been historically greatly underestimated when compared with the great emphasis placed on the left ventricle [LV] [6]. Meanwhile, it has been confirmed that RV assessment is of crucial importance for correct diagnosis, treatment, and prognostic evaluation of various CV disorders [6,7,8]. However, despite the important advances in understanding and managing RV dysfunction and right heart failure [RHF], many of the highly complex clinical challenges are still left to face [4,9,10]. In this regard, the assessment of both the RV adaptability to RVPO induced by an abnormal increase in pulmonary vascular resistance [PVR] and its ability for reverse remodeling (partial or even complete) and functional recovery after drug- and/or interventional-induced reduction in its PO towards the normalization of PVR has gained increasing interest, particularly in the fields of mechanical circulatory support [MCS] [11,12,13,14,15,16,17] and thoracic organ transplantation [18,19,20,21].

The aim of this article is to summarize current knowledge about the particularities of RV structural and functional responses to either acute or chronic RVPO induced by excessively high afterload and to provide an overview about the benefits and limitations of the currently available tools for the evaluation of RV adaptability to increased afterload in clinical praxis. A major focus of this article relates to the assessment of a potentially still present adaptability of the RV to pressure overloading and to evaluate its usefulness for certain critical therapeutic decision-making processes in intensive care units and in cardiothoracic surgery.

## 2. Particularities of the RV Adaptability to Hemodynamic Overloading

Ventricular adaptation to hemodynamic overloading, which refers to the ability to adjust both structure and function in response to pressure and/or volume overload (increased afterload and/or preload, respectively) in order to maintain the most optimal cardiac output [CO], is a vitally necessary cardiac response [22]. This response depends essentially on the primary involved ventricle, the type of overload (i.e., afterload or preload), as well as the degree and duration of overloading [22,23]. In addition, irrespective of whether only one or both ventricles are initially exposed to hemodynamic overloading, the ventricles can directly or indirectly affect one another during the adaptive cardiac responses aimed to prevent the reduction in CO [22].

### 2.1. Differences in the Adaptability to Hemodynamic Overloading Between the RV and LV

In healthy hearts, the RV and the left ventricle [LV] have very different load adaptability characteristics due to their distinct anatomical and functional particularities, according to their different roles in the CV system [4]. Where the LV is less tolerant to higher preload and can decompensate more rapidly during sustained volume overloading, but is at the same time particularly designed to handle the normally required higher pressures in the systemic arteries and can even withstand harmful increases in afterload (e.g., hypertensive crisis), the RV is much more volume tolerant, but is at the same time particularly less tolerant to sudden PO [4,10,24,25]. Patients with acute increase in their pulmonary vascular resistance [PVR], like in the setting of acute lung failure, often develop overt right heart failure [RHF] with a severely compromised CO, even in the presence of a rather moderate increase in RV afterload [24]. All these major differences between the two ventricles preclude the use of the skills and knowledges regarding the evaluation of the left-sided heart structure and pathophysiology also for evaluations of the right-sided heart structure and function [4].

Under physiological conditions, the major role of the RV is less in generating pressure, but rather in streamlining the continually changing volumes of venous return into a relatively stable stroke volume [SV], which will be ejected into the low-impedance pulmonary circulation [PC] with only about 25% of the LV stroke work [4,26]. By handling the fluctuating venous return, the enhanced RV compliance is particularly suited to accommodate changes in the inflowing blood volume [4]. However, this high compliance is hardly beneficial in patients with RVPO and can even hamper the required increase in RV systolic function [9]. Normally, the RV coupling to the PC, which enables an adequate and continuous blood flow in the pulmonary arteries, takes place in an energy efficient manner, facilitated by the low resistance to blood flow in the remarkably compliant blood vessels of the typically low-impedance and low-pressure PC [4]. In the presence of an abnormally high PVR, the initially systolic RV dysfunction will subsequently also trigger a diastolic RV dysfunction [9]. The latter arises mainly from the abnormally high filling-pressure-induced increase in RV stiffening associated with a relevant impairment of RV relaxation, which will impede the blood inflow to the RV [9,23,27].

In a pressure-overloaded RV without intrinsic myocardial alterations, the systolic dysfunction associated with RV dilation, increasing TR and clinical signs of RV failure [RVF] usually already occurs before the occurrence of irreversible alterations in RV myocardial contractility [4,9]. Especially in PAH, in a large proportion of patients with a high afterload-induced reduction in RV ejection fraction [RVEF], with and without additional signs of uncoupling between the RV and the pulmonary artery [PA], the RV contractility is usually increased rather than decreased before entering into the last stage of RVF [9,27,28]. Despite the high dependency on its afterload and its low tolerance to sudden pressure overloading, the RV shows a strikingly high adaptability to chronic pressure overloading [24,29,30,31,32]. In patients with PAH, even those with systolic pulmonary arterial pressure [sPAP], values of 85 mmHg can remain for a relatively long time, free of clinically manifested RVF [29]. In chronic PH, the RV can even face a 5-fold rise in afterload, which is considerably more than the about 50% increase that can be faced by the LV in patients with systemic hypertension or aortic stenosis [4,33]. In PAH patients, the RV end-systolic elastance [Ees] was also found to be about five times higher than normal, which indicates a strikingly high increase in the contractile state in the chronically pressure-overloaded RV [9,30,34]. All this can explain the ability of the RV for reverse remodeling and functional improvement in certain patients with RVF triggered by abnormally high post-capillary and/or pre-capillary PVR of different etiologies, after medical therapy- and/or device therapy-induced massive reduction in RVPO towards the normalization of the PVR [31,35].

### 2.2. RV Responses to Acute and Chronic Increase in Afterload

During RVPO, a great challenge for the RV is to remain coupled to the increased afterload [36]. In its proper sense, RV afterload is the RV wall stress that occurs during ejection mainly by the RV interaction with the PVR [37]. The adaptation of the RV to increasing afterload depends on RVPO severity, the duration of pressure overloading and the rate of its progression (e.g., chronic steadily rising or acutely occurring massive rise in resistance to pulmonary blood flow), as well as on the RV intrinsic myocardial contractility and, in this respect, especially on the myocardial adaptability to provide the necessary support for maintaining that overly high wall stress [4,11].

- RV responses to acute PO

Thanks to the intrinsic capability of the RV for short-term changes in myocardial contractility in response to sudden variations in end-diastolic muscle fiber length, acute RV myocardial overstretching promptly induces an increase in RV contraction force. Thus, acute RV afterload increases, able to reduce the SV, will be suddenly followed by end-diastolic myocardial distension (preload stretch), which instantly triggers a rise in myocardial contraction, aiming at preserving the SV [4,31,38]. Only a few minutes later, this transitory “heterometric” adaptation (i.e., Frank–Starling law) will be replaced by a short-term “homeometric” adaptive response (Anrep effect), characterized by a higher ability of the myocardium to enhance its contractility (independent of neurohormonal influences) in order to preserve the RV ejection volume without changing its chamber size (i.e., in the presence of a normalized end-diastolic volume [RV_EDV_]) and without increasing the RV filling pressures [2,4,31,38,39,40]. In the presence of acute massive RVPO (e.g., pulmonary embolism or acute respiratory distress syndrome induced by extensive pulmonary microangiopathy associated with small vessel thrombosis like in severe COVID-19 respiratory infections), if the homeometric adaptation becomes exhausted before it can be strengthened by RV hypertrophy [RVH], a heterometric adaptation is turned on again to preserve the CO, but this time at the price of RV dilatation and upstream congestion associated with a high risk of life-threatening acute RVF [7,39,41,42].

- RV responses to chronic PO

Chronic RVPO leads initially to an adaptive concentric RV remodeling characterized by wall thickening with higher myocardial contractility in the presence of an overall normal up to a slightly increased RV mass [4,22,31,43]. The higher contractility enabled by the upregulation of several subcellular organelles (sarcolemma, sarcoplasmic reticulum, myofibrils and mitochondria), simultaneously with a reduction in RV wall tension (thanks to the increased wall thickness in the presence of a preserved RV_EDV_), can prevent reductions in the SV in the early stages of RVPO [4,31,43]. In more severe chronic RVPO, the initial adaptative responses are enabled by a concentric RVH [31,44]. The latter, being characterized by a distinctly high wall thickening with a reduction in the RV cavity size which, by increasing the RV mass to volume [M/V] ratio, reduces the end-systolic wall tension and thereby allows the preservation of CO and exercise capacity, is usually associated with a higher RV wall stiffness and moderate diastolic dysfunction which initially, up to a certain level, are not clearly maladaptive [4,22,31,43,44,45]. Thus, in the initial adaptive phase, a moderate increase in fibrosis, together with an increase in cardiomyocyte stiffness, can provide support for maintaining RV shape and function [46,47]. However, with aggravation of a persistent PO, the increasing fibrosis contributes to RV diastolic stiffness and dysfunction, which hampers the passive emptying of the right atrium [RA] into the RV and will become associated with a reduction in patient survival [46,47].

Given the reduced adaptability of a normal healthy RV to sudden increases in PVR, a successful RV adaptation to high afterload without RVH is highly unlikely [4,48]. RV cardiomyocyte hypertrophy occurs through their increase in size, enhanced synthesis of additional sarcomeres and accumulation of sarcomeric proteins, usually accompanied by the re-emergence of a fetal gene expression pattern and a switch from α-myosin to β-myosin heavy chain, considered as a compensatory mechanism aimed at maintaining RV function in higher hemodynamic loading conditions [4,31,48]. However, in chronic RVPO, functional compensation does not preclude a RV maladaptive remodeling [22]. Thus, mitochondrial and sarcomeric dysfunction, as well as T-tubular loss, can already be present in the adaptive RVH phase, but are often hidden by opposing actions of certain hyper-functional cardiomyocyte components, such as increased Ca^2+^ sensitivity of myofilaments [22].

**Figure 1:**
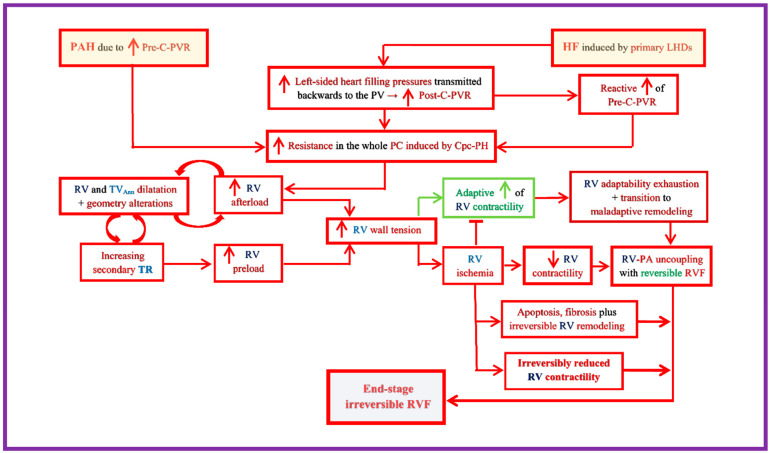
Summary overview of the major pathogenetic mechanisms underlying the emergence and development of RVF in the presence of a permanently increased resistance to blood flow in the PC [2,3,4,9,10,16,22,25,28,30,31,33,39,40,43,44,49,52] RV= right ventricle; RVF = RV failure; HF = heart failure; LHDs = left heart diseases; PAH = pulmonary arterial hypertension; Pre-C-PVR = pre-capillary pulmonary vascular resistance; PC = pulmonary circulation; Cpc-PH = combined post- and pre-capillary pulmonary hypertension; TV_Ann_ = tricuspid valve annulus; TR = tricuspid regurgitation. The arrows inside the boxes indicate increase (↑) or decrease (↓). Green lettering and green arrows inside or outside the box indicate favorable (adaptive) responses. 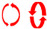 indicate a “vicious circle”.

Long-standing RVPO, especially if associated with further afterload increases, will finally induce a transition from the adaptive (compensate) RVH to a maladaptive RVH with increasing tendency towards functional decompensation [22,49]. The transition towards a decompensate phenotype often results in an eccentric RVH characterized by progressive spherical dilation of the chamber with concurrent wall thickness reduction towards an only moderate thickening, associated with myocyte loss, replacement fibrosis and dyssynchrony of contraction [23,43,50]. Factors generally associated with the transition from adaptive to maladaptive RVH also include ischemia and excessive neurohormonal chronic overactivation whose involvement is important regarding the development of RVF [31]. In patients with PAH, those with lesser RV hypertrophy and greater RV dilation (i.e., reduced M/V ratio) showed a high risk for cardiac events, already before the reduction in the cardiac index [CI] [43,44,51]. In patients with idiopathic PAH [IPAH] those with RV M/V values < 0.46 revealed significantly higher RV filling pressures, higher RA pressure [RAP] and lower CI values, as well as more impaired exercise tolerance compared with those having M/V ratios > 0.46, although there were no significant differences in PVR, in mean PAP [mPAP], as well as in PAC between the two patient groups [52]. Throughout maladaptive RV remodeling with and without myocardial hypertrophy, there are further stages of reversible and irreversible RVF and both adaptive and maladaptive phenotypes are also not completely different responses, but rather parts of a sequence of several RV responses to PO [30]. Currently, several mechanisms involved in the transition from compensated RVPO toward RVF are still incompletely elucidated [39]. An overview on the RV responses to PO and the major pathogenic mechanisms underlying the development of RVF is provided in Figure 1.

The fact that the overall risk of RVF and mortality in PAH is more linked to RV volume than to RV mass alterations, to PVR and mPAP values, as well as to LV morphological and functional changes occurring as a consequence of ventricular interactions, is a strong rationale for considering magnetic resonance imaging [MRI] as an important method in critical therapeutic decision-making processes [51,53]. More recently, it was also found that in PAH patients, the monitoring of RV_EDV_ changes alone can be helpful not only to evaluate RV adaptability to its hemodynamic overloading, but also to predict the worthening of PAH [51]. Thus, RV volume changes evaluated by MRI in association with changes in clinical status, over a period of 2.5 years, revealed that for a step change in WHO functional class, the associated change in RV_EDV_ was 11% (*p* < 0.0001) [51].

Although RVH and fibrosis occur in both mild and severe PH, recent studies indicated that certain fiber-level remodeling events (e.g., collagen fiber remodeling, increased tautness of collagen fibers and reorientations of myofibers) also contribute relevantly to the RV–PA uncoupling that, for its part, leads to RVF [45]. However, it remains unclear how and at what level the wall stiffening will become excessive and should be considered as maladaptive [45]. Reduced myocardial contractility, which in association with increasing RV end-systolic wall tension, reduces the SV, and the increasing secondary volume overloading of the right-sided heart, associated with increasing end-diastolic wall tension, finally lead to RV decompensation which compromises the function of the whole CV system [43,45]. In a study including 356 PAH patients divided into four different volume/mass groups, the 59 patients with a maladaptive RV remodeling referred to as “high-volume−low-mass” (i.e., overlay RV spherical dilation and lesser myocardial hypertrophy) revealed the lowest responses to therapy and the worst long-term survival [43].

Key biomarkers for right ventricular RVPO include natriuretic peptides (BNP, NT-proBNP) for wall stress and cardiac troponins (cTnI, cTnT) for myocardial injury [54,55]. Emerging, more specific markers include Cartilage Intermediate Layer Protein 1 (CILP1) and Heart-Type Fatty Acid-Binding Protein (hFABP), and Elabela (ELA) [55]. These also help assess RV function, prognosis, and remodeling in conditions like pulmonary hypertension [55].

### 2.3. Impact of the Left Ventricle on the RV Adaptability to Pressure Overloading

Basically, three types of interrelations (i.e., anatomical, functional and pathological) associated with a large degree of interdependence define the steady connection between the RV and LV during the development of PO-induced RV dysfunction [4,56,57,58]. The systolic and diastolic interactions between the ventricles enabled mainly by the interventricular septum [IVS], the pericardial space, and by certain myocardial tracts (epicardial circumferential myocytes) have important contributions to cardiac function in both healthy persons and in patients with different CV diseases [4,6,56,57]. In healthy persons, the LV contributes relevantly to the RV ejection and, in experimental studies, LV contraction generated between 20% and 40% of both RV systolic blood pressure [RVSP] and SV [4,6]. Based on their close and complex anatomical and functional interconnections, the pressure and/or volume overload affecting one ventricle can also affect the other ventricle, thereby impacting overall cardiac function [4,6,24]. Also, the remodeling of one ventricle can affect the contralateral ventricle [4,56,57]. In an initially healthy heart, if the RV is exposed to an abnormally high afterload, its pump function will be supported by LV contraction through their shared IVS, as long as the RV function remains compensated (without elevated RV filling pressures) [6,24]. However, being constrained by the pericardium, with the increase in RV end-diastolic pressure [RVEDP] which causes RV dilation, the latter will induce a leftward shift in the IVS and thereby an alteration of LV geometry, which distorts the normal interventricular geometric relationship and abolishes the functional support of the RV by the LV [6,24]. Thus, in PAH, due to the constraints of the relatively inflexible pericardial space and the consequent change in the transseptal pressure gradient during diastole, the dilated RV shifts the IVS leftward and alters LV geometry, thereby reducing both LV filling and its output [58]. In PH-LHD, the interactions between the pressure-overloaded RV and the damaged LV may vary greatly, depending mainly on the severity rate, the progression and the duration of the RVPO, as well as on the type of LV remodeling (eccentric or concentric) and on the presence or absence of pre-existing structural and/or functional RV alterations [56,57,59]. In contrast to the RVPO, in patients with pulmonary valve [PV] stenosis or in those with elevated pre-capillary PVR-induced PAH, where the RV responses can be improved by a healthy LV (mainly through IVS contraction), in PH-LHD, the functionally severely impaired and also structurally altered LV loses its ability to support the RV in overcoming the higher afterload in the PC [56,57,59]. However, given that the PC does not support a higher blood volume inflow than the volume that can be accepted by the left-sided heart and forwarded by the LV to the systemic blood vessels, an additional drug-induced improvement of RV pump function (e.g., reduction in pre-capillary PVR) without an at least equally efficient therapeutic support of the impaired LV would rather aggravate the pulmonary congestion [59,60]. This can also explain the higher incidence of PH-LHD in HF with preserved LVEF [HFpEF] compared to HF with a reduced LVEF [HFrEF] (i.e., 83% vs. 68%) and the notably higher probability of RV dysfunction in HFpEF, given that HFpEF is typically characterized by a small LV-EDV with more severely reduced SV [57]. All these can explain the lack of specific treatments to handle PH-LHD and to prevent the development of a severe biventricular decompensation apart from optimizing the treatment of the underlying etiology aimed to reduce LV dysfunction and to prevent a transition to irreversible LVF [59,60].

## 3. Reversibility of the Pressure Overloading-Induced RV Alterations

In the presence of a normal PV, the resistance that the RV must overcome to eject its SV into the PA is determined by the peripheric PVR (cross-sectional area and vascular tone), the PA compliance as well as its characteristic impedance, which reflects the pulsatile nature of the blood flow [5]. The prognosis and therapy results for patients with severe PH-LHD (which is the main cause of PO-induced RVF), as well as of those with the more rarely occurring but therapeutically particularly challenging PAH, depend on these two different PH groups with initially normal right-sided heart morphology and function essentially on the reversibility of the abnormally high afterload-induced maladaptive RV remodeling and dysfunction [17,20,21,61].

### 3.1. Reversibility of Chronic RV Failure Induced by Left Heart Diseases

After it has been repeatedly observed that in a large proportion of patients with advanced left-sided HF associated with PH-LHD and complicated by PO-induced RVF, the RV can still, for a long time, maintain its ability to respond with reverse remodeling and functional improvement to a medication and/or an interventional-induced reduction in its PO (reduction in PVR). This finding became particularly important for the evaluation of candidates for an MCS implantation, as well as for those necessitating a heart transplantation [HTx] [11,13,14,15,17,18,19,20]. In this regard, the prediction of a preserved RV responsiveness to the abolition of its steady PO by reversal of its maladaptive dilation has become a major goal with crucial impact on patient survival [62].

- Impact of high pre-capillary PVR in PH-LHD on decisions for or against HTx

HTx is the established treatment for eligible patients with end-stage HF, refractory to any medical therapy, as well as to any device and/or surgical optimization [63,64,65,66]. However, a major problem for the eligibility of patients with end-stage HF for HTx arises from the fact that HTx cannot be successful in patients with a LHF associated with irreversible structural and functional alterations in the PC able to cause insurmountable PVR increases [63,65,67]. Thus, in PH-LHD, as soon as the initial isolated post-capillary PH [Ipc-PH] turns into a combined pre- and post-capillary PH [Cpc-PH], the estimation of the reversibility of those structural and functional damages affecting the PC becomes crucial for the decision in favor or against HTx because the RV of the donor heart will be more than likely unable to overcome a persistently high pre-capillary PVR [40,65]. Thus, PH-LHD in which a reversal of the high PVR cannot be demonstrated is considered as a contraindication to HTx [63,65,68]. This challenging issue can become crucial because the prevalence of PH-LHD reaches about 26% in patients with end-stage left-sided heart-induced global HF [69].

- Impact of high pre-capillary PVR in PH-LHD on MCS therapy

During the last three decades, especially thanks to the growing clinical use of MCS devices, it has been proven that even severe secondary RVF induced by ‘fixed’ PH (i.e., medically unresponsive PH) can often be reversible after normalization or at least relevant reduction in RV afterload and therefore a durable LV assist device [LVAD] can offer the possibility for a bridge to candidacy for HTx [68,70,71,72,73,74,75,76]. In an early study, all six patients with end-stage HF and ’fixed‘ PH (average PVR 5.7 Wood units [Wu]) who received a LVAD as a bridge to a possible transplantability could finally be transplanted after a PVR reduction to 2.0 Wu during a mean support time of 6 months [70]. After a mean follow-up of about 16 months, five patients (83%) were still alive [70]. Two larger studies aimed to assess the ability of LVAD support to overcome fixed PH (i.e., mPAP > 25 mmHg, PVR > 2.5 Wu and transpulmonary pressure gradient [TPG] > 12 mmHg) and thereby to abolish the contraindication for HTx also revealed encouraging results [71,74]. Thus, in one study, 24 of 35 patients (68.6%), and in the other study, 19 of 27 patients (70.4%), became eligible for inclusion on the HTx waiting list during 6 weeks and <6 month of LVAD support, respectively [71,74]. Another evaluation of post-HTx outcome in 18 patients with pre-HTx reversible PH-LHD during LVAD support before HTx revealed no case of post-HTx RVF and the 30-day survival rate reached 100% [68]. Most notable were the results of a study in which using continuous-flow LVADs as a bridge to HTx candidacy, all 17 patients with ‘fixed’ PH-LHD already become eligible for HTx at the end of the 3rd month of LVAD [75]. Throughout the study period, nine of those patients bridged to HTx candidacy underwent HTx, which was successful in eight of them [75]. Given these promising chances for a relevant reversibility of high afterload-induced RV dysfunction, as well as the fact that the overall long-term outcome in patients after HTx is much better than after durable biventricular assist device [BVAD] implantation [62], the attempt to use a transitory LVAD as a bridge to a possible transplantability is undoubtedly worthwhile [63,68]. In trials evaluating the LVAD support with newer generation devices, the 2-year survival rate reached, on average, >80% a few years ago and is currently similar to that of HTx at 2 years, whereas even the 5-year survival rate of LVAD recipients has meanwhile reached nearly 60%, so that even if HTx remains ultimately not feasible, the durable LVAD support still remains an important therapeutic option [66,77,78,79]. In carefully selected PH-LHD patients who are not eligible for HTx because of irreversible alterations mainly in the pulmonary pre-capillary vessels (i.e., Cpc-PH with increasing TPG beyond 12 mmHg), the option of a combined heart–lung transplantation [HLTx] may also be considered [80,81].

Unlike PH associated with HFrEF, in PH associated with PH-HFpEF, the RV structural and functional alterations often appeared not associated with the degree of pulmonary vasculopathy; also, RV fibrosis often occurred out of proportion to the degree of RV afterload [80]. Also, unlike PAH patients, in those with PH-HFpEF, RV fibrosis appeared correlated to several indices of intrinsic RV myocardial remodeling, but not to the RV afterload [80]. This was also suggested by the observation that RV fibrosis and dysfunction were similar in PAH and PH-HFpEF despite the presence of a significantly lower RV afterload in the PH-HFpEF patient group [80]. These findings are of particular importance for the decision-making between a durable LVAD or BVAD implantation given that, although a LVAD is safer for patients and provides a better quality of life than a BVAD, a later vitally necessary implantation of an additional support also for the RV is usually much more risky than a BVAD implantation from the very beginning [62,82]. Despite the progress made in the area of MCS for patients with end-stage PH-LDH, several surgery-related events, as well as the hemodynamic implications inherent to left-sided univentricular support, can cause morphological and functional cardiac changes that may eventually result in early- or late-onset RHF [83,84]. In several studies, the RHF prevalence in LVAD recipients varied widely (between 5% and 44%), but there was always a close connection between RHF and both increased morbidity and higher mortality [84,85,86,87,88,89].

The RV-LV interactions in LVAD recipients also have certain particularities. Thus, an inadequate LV unloading (more often early after LVAD surgery) can induce severe RVF and it is challenging to recognize whether the RVF is mainly secondary (due to interactions of the supported LV with the RV and/or an insufficient reduction in the PVR) or primarily related to a deficient structural and/or functional RV adaptability to the hemodynamic overloading [79]. Therefore, a close monitoring of the RV and RA size, geometry and function, plus the assessment of TR changes in connection with the LV unloading by the LVAD became indispensable [83,84,90]. A recent study revealed that greater LV unloading by faster LVAD speeds induces increases in the RV_EDV_ concurrently with a decrease in end-systolic pressure, but without significant changes in RV contractility [91]. After LVAD surgery and gradually weaning the patient from the cardio-pulmonary bypass, a main goal is to balance LV unloading, systemic blood flow, and RV function. Thus, during LVAD speed adjustment, in addition to providing an optimal support for the LV ejection function, it is necessary to maintain LV filling in order to avoid a leftward IVS shift, because an excessive LV unloading-induced IVS shift alters the geometry of both RV and tricuspid annulus [TA] and also impairs the IVS contribution to the RV ejection function [79].

- Role of autoimmunity in PH-LHD

Given the important limitations imposed by the PH−LHD for medical and surgical treatment of advanced HF (e.g., increased risk of drug-induced vasodilation for developing pulmonary edema in LVF, detrimental consequences of ‘fixed’ PH on patient outcomes after HTx, limited possibilities and restrictive eligibility requirements for a combined heart–lung Tx), further therapy options for LV failure [LVF] appeared quite necessary. In this regard, the recognized essential role of altered immune responses in the pathogenesis of LV dysfunction induced by myocardial alterations that lead more often to LVF deserves much more attention [92,93,94,95]. Thus, the large group of non-ischemic cardiomyopathies, dilated cardiomyopathy [DCM], and especially the idiopathic and myocarditis-related DCM, which are among the more common causes for HF with PH-LHD, were found particularly strongly related to autoimmune disorders [69,93,94,96,97,98,99]. Chronic inflammation, detectable in about 50% of the hearts from patients with a DCM-induced HF, often appeared associated with high levels of autoantibodies [AABs] against both cardiac proteins and specific receptors (notably the AABs against β1-adreno-receptors [β1AR-AABs]), which are particularly relevant from a pathophysiological point of view [92,100,101,102,103,104]. The detrimental impact of the β1AR-AABs on cardiac cells is induced by a sustained activation of β1-ARs by stabilizing them in an active form [99,105]. The resulting overstimulation induces apoptosis and fibrosis, which can trigger a new-onset HF or aggravates the progression of an already existing HF [103,105]. Given that DCM is the leading indication for both HTx and LVAD insertion, many studies have focused on possible solutions for removal of cardiac AABs particularly in patients with DCM [99,102,106,107,108,109]. Abnormally high levels of so-called “functional” β1AR-AABs [β1-FAABs] (identifiable by bioassays of cell cultures with spontaneously beating neonatal rat cardiomyocytes), which appeared related with the severity of HF, were detectable in 84–97% of patients with end-stage DCM, and those β1-FAABs were effectively removable (below the pathological level) by immunoadsorption [IA] in about 80% of those FAAB positive patients [94,102,105,106,107,108]. Removal of the β1-FAABs appeared related to the improvement of LV and global heart function, as well as with a 5-year HTx/LVAD-free survival probability of 63–69% and was found able to spare many initially β1-FAAB-positive patients from HTx, or at least to delay the HTx listing for many years [99,102,105,108]. Although IA is time consuming and costly, it is less expensive than HTx or LVAD insertion and, because of its relative safety and good tolerability even for elderly persons, as well as the possibility to repeat the IA in case of AAB reappearance, IA therapy deserves much more interest, given that the post-IA 3-year HTx-/mechanical support-free survival probability can reach about 80% [99,105]. In the guidelines of the American Society of Apheresis on the use of therapeutic apheresis in clinical praxis, IA achieved a “grade B” (strong) recommendation with the remark that it can be used without reservation in most patients in most circumstances [110]. DCM is also graded as a “class II” disorder for which IA is recommended as second-line therapy either as stand-alone treatment or in combination with other treatment methods [110]. A particular problem which is still not completely clarified relates to the standardization of the methods necessary for the detection and quantification of cardiac AABs [99,105]. Thus, because not all detectable AABs are functional (i.e., able to bind and modulate the function of their target antigen) and thereby, the mere presence of an AAB type is not a guarantee for its pathogenic effects, it is important to establish whether the detected AABs are functional or non-functional [94,99,105,106,111]. Table 1 provides an overview of the available IA techniques which allow the removal of pathological cardiac AAbs involved in the pathogenesis of PH-LHD.

An alternative to IA can be the AAB neutralization by intravenous injection of small soluble molecules, such as peptides or aptamers, which can specifically target and effectively neutralize certain AABs like the β1-AABs [99,105,111,112]. Whereas peptides may induce immunogenicity and cytotoxic effects, the aptamers, which are nucleic acid molecules synthesized chemically in vitro (thereby being non-immunogenic), have so far not revealed safety concerns [105,112]. Aptamer synthesis, which is simpler and less expensive than antibody production, also ensures high reproducibility and consistency [105,112]. However, their potential implementation in the clinical praxis is currently still in the exploratory/early phase and further clinical trials are still necessary in order to assess the utility and safety of this relatively new approach to treat HF and thereby also PH-LHD [112].

### 3.2. Reversibility of Chronic Right Ventricular Failure in Pulmonary Arterial Hypertension

PAH is a progressive pulmonary vascular disease characterized by a high PVR induced by ongoing structural and functional alterations in the distal pulmonary arteries triggered by a diffuse obstructive and proliferative pulmonary arteriopathy, concurrently with increased fibrosis and a decrease in the compliance of the proximal PA walls, which will sooner or later be followed by a pre-capillary PO-induced RVF with increasing tendency for cardiac decompensation [39,45,113]. In about 50% of cases, PAH is idiopathic and, in spite of all the therapeutic advances during the past decades, IPAH remains, from a certain level onwards, still incurable [39,114,115,116,117]. In advanced PAH, for patients without absolute contraindications for LTx who did not respond to medical therapies, a double lung transplantation [DLTx] often remains the only therapeutic option [39,114,115,116,118,119]. Although making a too early decision for LTx is risky, given that LTx carries higher risks than other organ transplantations, a too long delay of a vitally necessary DLTx can become even more risky because, over time, the RV loses its ability to recover sufficiently even after normalization of the PVR and thus, a beneficial DLTx will become increasingly unlikely [118,119]. In an earlier study, among 59 listed patients, 24 (40.7%) died before LTx after less than 3 months and, even in those treated with iloprost and/or bosentan, the mortality on Tx lists reached 33.3% [114]. A recent multicenter analysis of patients with PAH came to the conclusion that the rates of referral for LTx are still low and more than often occur too late, even at expert centers [119]. The fact that about 20% of patients referred for DLTx died before Tx indicated that their referrals were made too late [119]. In end-stage PAH with severe PO-induced RV structural and functional alterations, a combined HLTx should also be considered because its long-term results are usually similar to those after DLTx, whereas in the case of a post-DLTx with the necessary long-term mechanical support of an already exhausted RV, the highly possible complications would clearly be more detrimental to the patient [119,120,121,122]. Pathologic immune responses play a major role in the pathogenesis of PAH and there is evidence that AABs against α1-adrenergic receptors [α1-ARs] and endothelin-1A receptors [ETARs] are most relevantly involved in the pathogenesis of IPAH [123,124,125,126]. In an earlier study, 51 (94%) of the 54 tested patients with PAH were found positive for FAABs against α1-ARs and, among them, 38 (75%) had FAABs against both α1-ARs and ETARs [123]. The FAAB induced ongoing stimulations without desensitization of the receptor-mediated signal cascade and first attempts to remove those AABs by IA revealed promising results [123]. The first IA therapy of five patients with end-stage IPAH associated with high levels of α1-AR-FAABs alone or together with additional ETAR-FAABs in their serum suggested that a removal of these FAABs can avoid the necessity for a DLTx [127]. After the completion of the IA-therapy cycles, the FAABs disappeared from the sera of all five patients and at the end of the 3rd post-IA week, all patients revealed a relevant PAP reduction associated with an improved RV function and an increased exercise capacity [127]. Thereafter, three of those patients became once again highly symptomatic at 3, 18 and 24 months after the IA, respectively, and the clinical worsening coincided with a redetection of the same FAABs in their sera [127]. After a second IA therapy in two of those three patients, one recovered again and remained thereafter stable over years [127]. The two women without a reappearance of FAABs after the first IA showed a relevant and stable clinical improvement and remained at NYHA-class II during the whole evaluation time of 4 years [127]. During all IA therapies, there were no relevant adverse events and all the therapy cycles were well supported by all patients, which indicates that AAB removal by IA can be safe and well tolerated for the patients [127]. In another study, 10 AAB-positive IPAH patients underwent 5 days of IA; the AAB removal also appeared to be safe and was associated with significant beneficial hemodynamic effects in seven of those patients [128]. At 3 months after IA, on average, the PVR decreased by 13% and the CI increase reached 13.3% on average [128]. Patients with the highest AAB levels at baseline revealed the most relevant reduction in PVR [128]. At the ninth post-IA day, the α1-AR-AAB, ETAR-AAB, and angiotensin II type 1 receptor antibody [AT1-AAB] levels were on average 48.4%, 23.1% and 35.6% lower than before IA, respectively [128]. Completely unexpectedly, despite the significant reduction in the PVR associated with significant increases in the CI during the first 3 months after IA, the initially reduced serum AAB concentrations increased again and at 1 month after IA, already reached values which, on average, even exceeded the baseline AAB levels [128]. Thus, whereas in the above mentioned study [127], only one of the five patients revealed a reappearance of FAABs associated with clinical worthening at 3 months after IA, in this study [128], a post-IA reappearance of AABs was detected in all 10 patients already at 1 month, although their IA-induced PVR reduction and RV functional improvement remained stable during the entire observation time of 3 months. A plausible explanation for those partly contradictory results could be the fact that only the first of the above mentioned two trials exclusively measured the FAABs [127] and therefore it is quite possible that in this second study [128], the reappearance of high AAB levels early after IA, despite the lack of any signs for a hemodynamic worsening, was caused by an increase of “non-functional” AABs. This underlines the importance of the distinction between non-functional and functional cardiac AABs.

Regarding the necessity to focus the therapeutic approaches much more on the pathobiological particularities of the PAH and, in this context, especially on the immunological factors involved in PAH, a recent FDA-authorized study revealed that heart-derived progenitor cells with anti-inflammatory and immunomodulatory effects (like cardiosphere-derived cells [CDCs]) could be notably useful [129,130]. The inclusion of CDCs into the PAH therapy could be particularly helpful to break certain vicious circles which are relevantly involved in the complex and multifaced pathobiology of that disease for which there is no cure, given that the approved PAH-specific medications are mainly pulmonary vasodilators with the potential to reduce cellular proliferation [118,130]. In the Phase 1 study, the intravenously injected CDCs to PAH patients had proved to be well tolerated and, in addition to their long-term safety, their significant positive effects on hemodynamics, pulmonary diffusing capacity, RV volumes’ reduction and functional improvement, as well as on the exercise capacity of the patients, appeared to be in all very encouraging [130]. As in the case of the aptamers, the potential implementation of this relatively new approach in clinical praxis is currently still in the exploratory/early-phase of its validation and further clinical trials are absolutely necessary in the near future in order to confirm its clinical utility and safety for PAH therapy.

## 4. High RV Afterload-Induced Alterations in Its Pressure Balance with the RA

Given the high variability of TR severity in RVPO and its various impacts on the interrelations between the RVSP and the RAP, their direct measurement by right heart catheterization [RHC] as well as their non-invasive estimation by echocardiography [ECHO] are essential for evaluations of the PO-induced RVF [131,132]. In this regard, of high practical relevance is the Doppler-ECHO-derived TR pressure gradient [TRPG] (abbreviated also as TRG), which represents the difference between the RVSP and RAP [ΔP_RV-RA_]) [131]. However, none of these major hemodynamic parameters enable a reliable severity grading and/or prognostic estimation of right-sided heart dysfunction, neither individually nor in combination with one another [132]. Thus, in the presence of a normal RV function and TR ≤ 1, the transthoracic ECHO [TTE]-derived TR maximal velocity [TR_Vmax_] and the calculated TRPG are below 2.9 m/s and 34 mmHg, respectively, and in severe TR these parameters increase up to >3.6 m/s and ≥52 mmHg; in “wide-open” (i.e., extremely sever) TR caused by the right-sided heart dilation-induced displacement of TV leaflets, the TR_Vmax_ and TRPG values can even misleadingly drop below 2.5 m/s and 25 mmHg, respectively [132]. Thus, two different persons, one with normal RVSP and RAP values (e.g., 30 mmHg and 5 mmHg, respectively) and another with a high RVSP associated with high RAP (e.g., 40 mmHg and 15 mmHg, respectively), can have misleadingly identical ΔP_RV-RA_ values (in this example, 25 mmHg). This is explainable by the fact that in PO-induced RVF with secondary “wide-open” TR, the RV and RA can essentially behave like a “single chamber”, so that the ΔP_RV-RA_ can become lower than in patients with less severe TR and also lower TR than in healthy persons [132,133]. This suggests that even correctly measured RV and RA pressures may alone become insufficient and even misleading for evaluations of both RV dysfunction and TR severity, as well as for the assessment of RVF prognosis in certain patients with PH-LHD or PAH associated with refractory wide-open TR [132].

## 5. Non-Invasive Estimation of RV Pressure Overloading

Given the increasing consideration of the role of TTE in the evaluation of the right heart in patients with PH, the American Society of Echocardiography has recently updated their guidelines from 2010 accompanied by excellent echocardiographic discussions [134]. Among the most important non-invasive screening parameters for PH overall is the TTE Doppler-derived TRPG (peak RV–RA pressure gradient) which is estimated from the TR_Vmax_ by using the formula TRPG ≈ 4 × (TR_Vmax_)2 and the mean RV–RA pressure gradient obtained from the velocity–time integral of the TR [VTI_TR_] [132,135,136]. Because of the low-pressure gradient across the PV and the outflow tract, as long as the PV is normal and there is no stenosis in the proximal PA, the RVSP and the sPAP are nearly the same. Thus, non-invasive screening for the detection of a high PVR, both the TRPG and the estimated sPAP (i.e., TRPG + RAP), can be used. However, given that the ECHO-derived RAP estimation is limited by a low accuracy, the sole use of TRPG could be more reliable in the presence of optimal Doppler signals [136]. One study analyzing the reliability of the non-invasive PAP assessment by two-dimensional [2D] ECHO compared to RHC-derived PAP measurements has confirmed the importance of optimal Doppler signals, given that the most common cause for underestimations of sPAP values was the presence of an incomplete spectral wave envelope [131]. Another cause for the underestimation of sPAP was the presence of a severe TR [131]. Also in that study, among the patients which had to be excluded due to a too small (i.e., not evaluable) TR, in about 53% of them, the RHC nevertheless revealed the presence of a PH, which confirmed that the absence of a measurable TR does not exclude a PH [131].

Given all the limitations of the ΔP_RV-RA_ estimations from TR signals, the RV outflow tract acceleration time [RVOT-AT], measurable on the pulse-wave Doppler signal obtained proximal to the PV (i.e., the time from the start of ejection to the peak flow velocity), has gained a growing importance especially for its use in persons with an inadequate TR jet [131,137]. Whereas values of >130 ms are normal, values < 100 ms are highly suggestive of PH or PAH. Overall, the RVOT-AT showed the highest sensitivity for the prediction of PH in patients with low TRPG and is usually available if the TR jet is insufficient. It was also found to be the best ECHO-derived screening parameter for elevated PVR (≥3 WU) [136,138]. The RVOT-AT also allows the calculation of the mPAP using the formula: mPAP = 90 − (0.62 × RVOT-AT) [136]. The TRPG/RVOT-AT ratio is also useful and, in certain patients, that ratio appeared to be even more useful than the individual use of TRPG and RVOT-AT [136].

## 6. Assessment of Pressure Overloading-Induced RV Alterations

RV function is ultimately the most important predictor of patient outcomes with PH [139]. In patients with “aberrant RV”, it is basically necessary to establish whether the underlying cause is a pressure or volume overload, or rather a primary myocardial process, because the progression of the disease and the therapeutic approaches in these three clinical situations differ accordingly [4,140]. In patients with evidence of anatomical and functional RV alterations concomitantly with a high resistance to blood flow in the PC, the major goals of RV assessment are the severity grading of the RV alterations and the evaluation of the causal relationships between certain right-sided heart changes and the RVPO severity [32].

### 6.1. Non-Invasive Methods for Assessment of RV Alteration

To assess the right-sided heart for the possible detection of relevant morphological alterations, as well as its systolic and diastolic function aimed at the timely detection of relevant dysfunction and making the most appropriate therapeutic decisions, non-invasive evaluations using TTE as a first-line tool, and both cardiac magnetic resonance [CMR] and computed tomography [CT] as advanced diagnostic modalities appeared most useful [4,140,141,142,143]. In addition, both nuclear imaging and positron emission tomography [PET] can provide useful functional details [142], whereas 3D-ECHO and certain laboratory tests can also offer valuable supportive data [144]. The limitations of using those advanced diagnostic modalities can especially be the lesser availability and patient suitability for CMR, the risks of using gadolinium CMR for patients with chronic kidney diseases (risk of acute kidney injury), as well as the radiation exposure (rather relatively low) during CT [145,146,147]. However, CMR is the gold standard for RVsize and EF quantification [147].

### 6.2. Assessment of Pressure Overloading-Induced Morphological RV Alterations

TTE is essential for the initial diagnostic clarification of suspected RVPO [32,134]. This is possible thanks to its ability to provide valuable details on RV and RA size and geometry, RV wall thickness, IVS position and displacement, size and morphology of right-sided heart valvular rings, as well as on the morphology and mobility of valve leaflets [32,140,148]. Thus, the elevation of the end-diastolic RV/LV diameter ratio in the apical four-chamber view and that of the end-systolic RV eccentricity index [EI] (calculated from RV dimensions measured in the parasternal short-axis view) are both key indicators of RVPO provided by 2D-ECHO [32,134,140]. However, because 2D-ECHO offers only planar imaging, quantitative assessments of the chamber volumes, as well as the quantifycation of RV mass are not reliably possible [32,140]. Regarding this, three-dimensional [3D] ECHO is more reliable for assessment of the RVPO-induced morphological alterations of the right-sided heart, as well as their relationship to the morphological alterations of the left-sided heart [149]. One major advantage of the 3D- over the 2D-ECHO is the much more reliable quantification of heart chamber volumes [4,32,140,148,149]. Transesophageal ECHO [TEE] is hampered by the limited movement of the transducer in the esophagus and the position of the RV in the far field and can therefore not represent the real size of the RV [150]. TEE (especially real-time 3D-TEE) can be particularly beneficial for the evaluation of the RV size, geometry and volume in intensive care units for patients with poor TTE image quality, or in the operation room, especially during and early after LVAD implantation [151,152].

CMR is the standard non-invasive imaging tool for visualizing and quantifying the cardiovascular anatomy, including the heart chambers volumes [141,153,154,155,156]. By providing more detailed anatomical images, CMR is particularly important for accurate estimations of the RV myocardial mass and the volumetric calculation of the RVEF, as well as for the best possible assessment of the tricuspid valve [TV] plane [141,142,144,154,156,157].

Due to its high spatial resolution plus its ability for volumetric data acquisition, CT is useful for the assessment of cardiac morphology (especially for measurements of the TV annular dimensions, RV and RA chamber dimensions and their wall thickness) [140,143,154,158]. By using CT in combination with a semiautomatic 3D hybrid segmentation approach, a highly reliable RV mass quantification also becomes possible [159]. CT pulmonary angiography is also suited for assessing cardiac chambers and signs of RVF by allowing a better detection of a flattened or bowing IVS and a widened RV, but its use is limited by certain adverse side effects [160].

### 6.3. Assessment of Pressure Overloading-Induced Functional RV Alterations

Assessment of RV function, with or without direct inclusion of its morphological characteristics, is even more challenging than the purely anatomical assessment of the RV, particularly regarding the interpretation of functional parameter alterations concerning the relative contribution of the often secondarily impaired myocardial contractility by the PO-induced RV dysfunction [32].

2D-ECHO is useful for routine evaluations of RV function especially by enabling the assessment of the lateral TA peak systolic excursion [TAPSE] and RV fractional area change [FAC_RV_] which both do not require geometric assumptions and which facilitate the detection and close monitoring of RV dysfunction [6]. The TAPSE has the advantage of being measurable even in the presents of a limited image quality [4,32]. Yet, it should be taken into account that both parameters are load-dependent and, in addition, TAPSE measurements are also highly angle-dependent [4,32]. Another limitation of TAPSE is the simplification of its diagnostic value in assuming that displacement of a single wall segment can reliably reflect the contractile function of a complex 3D structure [32,134]. It should also be taken into consideration that TAPSE does not include the contribution of the IVS and the RVOT to the overall RV systolic performance [32,161]. Often neglected is the fact that TAPSE can be relevantly and also misleadingly affected by the LV function and especially by the overall motion of the heart during systole [32,134,150,162,163,164,165]. The impact of the rocking motion of the heart in severe PH with a dilated and dysfunctional RV, where the LV longitudinal rotation pulls the TA down passively, can also misleadingly affect the TAPSE value [162,163]. In a study aimed to quantify RV function in patients with thromboembolic chronic PH before and after pulmonary endarterectomy [PEA], although several hemodynamic and clinical parameters, as well as the FAC_RV_ and certain 2D-ECHO-derived tissue Doppler imaging [TDI] parameters improved significantly after PEA, the TAPSE revealed often misleading biphasic responses [163]. Thus, whereas at baseline, the TAPSE appeared enhanced by the rocking motion of the failing heart (14.5 mm preoperatively), postoperatively the TAPSE drops astonishingly, reaching only 8.5 mm after 1 week and still remaining 24% lower than preoperatively at 6 months [163].

Regarding the reliability of FAC_RV_, this can be affected by a suboptimal endocardial definition due to the heavily trabeculated myocardium mainly during systole [32,166]. Like TAPSE, FAC_RV_ does not include the contribution of the RVOT to ejection and, in the presence of TR, both parameters will progressively overestimate the RV pump function given that they do not account for the blood volume flowing backward into the RA during the RV systole [32,161,167]. Despite those limitations, a strong association was found (*p* < 0.001) not only between the 2D-TTE-derived end-diastolic area (RV_EDA_) and the MRI-derived RV_EDV_, but also between MRI-derived RVEF and the two 2D-TTE-derived functional RV parameters FAC_RV_ and TAPSE [168]. This suggests that, taking their limitations into consideration, certain TTE-derived RV parameters can be used for routine RV monitoring in order to facilitate an early detection of additional RV alterations which necessitate further clarification. However, due to its insufficient reliability for measurements of RV volumes, 2D-ECHO is not anymore recommended for estimations of the RVEF [32,134].

The 2D-ECHO-TDI is also helpful for assessments of the RV contractile function [32,134,161]. A limitation, in addition to the angle dependency of TDI measurements, is the fixed position of the sample volume which does not enable the tracking of the surveyed area throughout its movement during the cardiac cycle and with respiration [32]. Nevertheless, parameters like the lateral TA peak systolic motion velocity [TAPS’], the RV myocardial isovolumic contraction peak velocity [IVCV] and the RV isovolumic acceleration [IVA], defined as the ratio of IVCV to the time-to-peak velocity, appeared useful for assessment of RV systolic function [32,150,169]. An advantage of IVA is its lower afterload dependency [32]. However, the lack of validated reference values and especially the misleading effect of TR on RV-IVCV can limit its practical use [32,150]. IVA measurements also appeared dependent on patient age and heart rate [32,133].

A major limitation of common ECHO for evaluations of ventricular pump function is its inability to distinguish between active and passive movements of certain parts, like the IVS leftward passive motion during systole that is not caused by its myocardial contraction, but rather by an early rise in RV pressure, creating a pressure gradient that pushes the IVS into the LV [56,170]. Another example is also the traction of the RV free wall [RVFW] at the points of attachment to the LV, induced by the isovolumetric LV contraction, which causes the RVFW to bulge passively into the RV [32,166,171]. Over the course of the last two decades, the limitation of ECHO to differentiate between active (myocardial contraction-induced) and passive movement of different RV regions has been successfully overcome by the implementation of speckle-tracking ECHO [STE] [32,165,171,172,173,174,175,176,177]. The STE, by its ability to assess myocardial deformation (also referred to as strain [S]), a parameter which is unaffected by the motion of the entire heart or that of myocardial regions (e.g., IVS), allows not only quantifications of the S magnitude and its velocity (referred as strain rate [SR]), but also the distinction between active and passive movement of the myocardial tissue in different ventricular wall segments [164,171,172]. The fact that in patients with PAH, conventional parameters of RV systolic function like TAPSE, FAC_RV_, TAPS’ and RVEF can be normal despite abnormal RV strain and/or SR values, explains the growing clinical usage of S and SR parameters particularly in these patients [173]. The angle independency of S and SR measurements is another important advantage of the STE [164,174]. The global RV longitudinal peak systolic S and SR ([RVGLS] and [RVGLSR], respectively), as well as the RVFW peak systolic longitudinal S and SR ([RVFWS] and [RVFWSR], respectively), revealed significant prognostic values regarding the risks of CV morbidity, aggravation of RVF and of higher mortality in patients with PH and PAH [173,175,176,177,178]. In PH of different etiologies, the RVGLS appeared closely correlated with the FAC_RV_ and also with several RHC-derived pulmonary hemodynamic measurements and, in contrast to the TAPSE, both RVGLS and FAC_RV_ also revealed significant predictive values for 5-year mortality [177]. However, only the RVGLS also revealed a significant association with hospitalization [177]. The peak systolic longitudinal S of the basal RVFW also revealed a high sensitivity and specificity for preoperative prediction of RVF after LVAD implantation [178]. In other studies, the STE-derived S and SR parameters appeared also useful for the evaluations of RV intraventricular dyssynchrony [32,119,179].

There are three main contraction mechanics involved in the RV pump function: (1) longitudinal axis shortening with traction of the TA towards the apex, primarily induced by the contraction of the sub-endocardial longitudinal myocardial fibers; (2) radial shortening by the inward movement of the RVFW (also termed as “bellows effect”) driven by the circumferential fibers from the subepicardial layer, often being enhanced by LV contraction; and (3) anteroposterior reduction in the RV diameter, induced by both bulging of the IVS into the RV and stretching of the RVFW during contraction of the LV (as part of the “bellows effect”) [30,36,133,166]. The assessment of RV systolic function revealed that, whereas the longitudinal and anteroposterior components of RV contraction decrease in the early stages of LV dysfunction, the radial component increases to preserve RV ejection function [149]. ECHO parameters, which refer only to the RV longitudinal shortening (i.e., TAPSE and TAPS’), can underestimate the RV systolic function because in the presence of left-sided heart diseases, the radial shortening improves initially, aiming to provide a functional compensation [149]. It is therefore quite comprehensible that 2D-ECHO-derived parameters are hardly capable of providing reliable details about certain morphological and functional RV alterations and, in this respect, 3D-ECHO may have several important advantages [36,149,165,180]. Thus, supported by software solutions which optimize the estimation of RV geometry and improve the measurement of RV volumes, the 3D-ECHO also allows volumetric calculations of the RVEF [36,165]. Novel postprocessing solutions for more detailed analyses of RV shape and function, as well as optimizations of 3D-STE, which allow the separate estimations of the different myocardial motion and deformation components (longitudinal, radial, and anteroposterior), enable the 3D-ECHO, in many aspects, a better evaluation of the RV alterations in comparison to 2D-ECHO [36,149,181].

By enabling accurate measurements of RV volumes for the calculation of RVEF, the 3D-ECHO is particularly useful. However, it should not be forgotten that the volumetric calculation formula, RVEF% = [(RV_EDV_ − RV_ESV_)/RV_EDV_] × 100%, is only valid if RV_EDV_ − RV_ESV_ is truly the ejected blood volume into the PA [182,183]. This often neglected aspect is important because a relevant RVPO is frequently accompanied by relevant TR [156], which impairs the validity of the calculated RVEF because the difference between the RV_EDV_ − RVE_SV_ will become the sum of RV forward SV [fSV] and the regurgitant volume [RegVol], thereby inducing a misleading overestimation of the RVEF [182,183]. This limitation can be easily overcome by using the formula fRVEF% = (fSV/RVEDV) × 100% for patients with TR, where fRVEF is the so-called “forward RVEF” [181], a new functional RV parameter which was recently also referred to as “effective RVEF%” [eRVEF] [183,184]. By combining the 3D-ECHO-derived RV volume measurements with the Doppler-derived tricuspid RegVol value, the eRVEF can be calculated by subtracting the RegVol from the “total RV stroke volume” to obtain the fSV (i.e., fSV = [RV_EDV_ − RV_ESV_] − tricuspid RegVol) which, divided by the RV_EDV_, will yield the eRVEF (i.e., eRVEF% = [fSV/RV_EDV_] × 100%), a synonym with exactly the same meaning as the fRVEF [183]. As expected, in patients with secondary TR, the eRVEF was found to be more closely associated with all-cause mortality and HF hospitalizations than the conventional RVEF and other ECHO-derived indices of RV function [183]. This finding attests the prognostic value of eRVEF for the estimation of RV systolic function in patients with TR [183]. Given the often misleading impact of TR not only on the evaluation of the RV functional parameters, but especially its steadily growing contribution to the aggravation of RV dysfunction, the assessment of its severity, as well as the estimation of its prognostic impact on individual patients, should be a major goal. In this regard, the 2D-ECHO can be often sufficient for a routine monitoring of the TR course [157,185]. However, given that standard 2D-ECHO often underestimates the true severity of TR, for decision-making regarding a possible transcatheter TV therapy, the use of a 3D-ECHO will be necessary [185,186,187]. In such cases, a multiparametric ECHO approach to grade TR severity is indispensable [188,189,190].

CMR is widely acknowledged as the standard for the non-invasive evaluation of cardiac volumes and function [155,184]. Due to its ability to allow, in addition to accurate quantification of RV volumes, reliable measurements of wall thickness, estimations of RV mass plus the assessment of major RV functional parameters, CMR is necessary not only for clinical research, but also in clinical praxis for special therapeutic decisions [152]. In patients with TR, a comparison between the eRVEF and the conventional RVEF (both calculated from CMR-provided RV volume data) revealed a relevant incremental benefit of eRVEF over conventional RVEF to predict mortality and HF events [184]. CM can also quantify the TR severity by using special techniques like phase-contrast imaging [191].

CT can be used for assessment of RV function by allowing RV volume measurements necessary for the calculation of RVEF [160,191]. Given that CT uses X-rays and also often contrast agents, and that it also overestimates the RV volumes and EF compared to CMR, CT is not a first-line tool for the RV [191]. However, CT can be a useful alternative when ECHO provides insufficient information or if CMR is either unavailable or contraindicated [191]. CT is in many cases superior to 3D-ECHO, being particularly helpful for obtaining specific anatomical details necessary for a detailed individual pre-procedure planning of TV repairs (especially for TA dimensions, coaptation gaps, and relationships to the coronary arteries) [192,193].

## 7. Integrated Multiparametric Approaches for Evaluation of RVPO

Early detection of PO-induced RV alterations, their severity grading, the estimation of their prognostic impact, as well as the close individual monitoring of their time course, are indispensable for making the most appropriate early therapeutic decisions in order to prevent or at least delay as much as possible the transition to irreversible RV damages [32,134]. However, the simultaneous presence of a distinctively high sensitivity of RV size, geometry and function to PO and a higher RV tolerance to volume overloading can have relevant misleading effects on the reliability of any single parameter used in RV evaluations [4,9,40,194]. In this regard, it is important to consider the fact that the evaluation of the RV depends not only on the used techniques and the measured parameters, but also and often on the interpretation of the measurements in the context of the specific features and the presently existing RV systolic and diastolic loading conditions [194,195,196,197,198]. It is therefore not surprising that single parameters cannot reliably reflect RV function or even predict its prognosis across the spectrum of RVPO [32,134]. Because even the myocardial deformation parameters, which enable direct quantifications of the contraction-induced changes inside the RV walls are load-dependent, all measurements need to be interpreted in conjunction with the present RV hemodynamic loading conditions. Thus, an increase in RV afterload can reduce the RV strain values, even if the intrinsic RV myocardial pump function remained preserved [134]. Usually, only integrated multiparametric approaches combining parameters of both RV structure and function, all evaluated in relation to the current hemodynamic overloading, can become sufficiently reliable for diagnosing and prognosticating the PO-induced RV dysfunction [32,62,134]. Among the most challenging tasks in making vital therapeutic decisions are those related to the indication and the optimal timing of certain highly life-saving surgical therapies for severe chronic RVF induced either by end-stage left HF, or by an advanced therapy-resistant IPAH. In such critical situations, a reliable prediction of a still preserved RV responsiveness to a surgical therapy-induced abolition of the steady RVPO by reversal of both its maladaptive remodeling and its severe dysfunction can be decisive for patient survival [14,31,152]. Table 2, which provides an overview of the main goals related to the right-sided heart assessment in persons with ongoing PO-induced RV dysfunction caused by high post-capillary and/or pre-capillary resistance in the PC, as well as the ways of achieving those objectives, reveals the complexity of this task [16,17,31,40,43,53,56,83,142,152].

For non-invasive evaluations, using TTE is the first-line tool for initial diagnostic clarification of suspected PO-induced right-sided heart anatomical and functional alterations, usually without the primary necessity for additional inclusions of RHC-derived measurements [11,15,20,64,152,199,200,201,202,203,204,205,206,207,208,209,210,211,212,213,214,215]. Mainly for the RV, it is necessary to evaluate the size, geometry and the pump function in relation to its current loading conditions (first of all with its afterload) [216].

Because of its simplicity, safety and efficiency, the TTE is the preferred non-invasive method for the detection and severity grading of PO-induced RV dysfunction, for the prediction of patient outcomes and for the monitoring of patient responses to therapy [134,137]. Although many reports have confirmed the suitability of TTE for right-sided heart evaluations, the complex and also highly variable interactions between certain structural and functional changes in both the hemodynamic overloaded right-sided heart and in the PC can often change the reliability of certain ECHO-derived parameters in the course of the disease [213]. In this regard, among the most crucial problems are those related to the detrimental impact of a secondary TR aggravation as a consequence of the TV annulus enlargement and its geometry changes induced by the dilation of the right-sided heart cavities [29,213,217]. Thus, severe TR has, in addition to its deleterious impact on RV function and its misleading impact on the diagnostic relevance of the TAPSE, a misleading negative impact on the accuracy of the TTE-derived estimations of both the sPAP and the RAP [206,213,217,218]. Many studies revealed significant differences between ECHO- and RHC-derived sPAP values with more than 50% mismatch rates especially in severe PH with advanced RV dysfunction and severe TR [217]. Thus, the TTE-derived TAPSE/sPAP ratio will also become less accurately estimable in patients with severe TR [206,212,213,214,217]. In patients with optimal TR-derived Doppler signal quality, the overestimation of sPAP usually becomes relevant in the presence of TR with a vena contracta [VCTR] width > 7 mm and affects most of the sPAP estimations in patients with a VCTR width > 11 mm [206]. A study investigating the impact of severe TR on the reliability of TTE-derived sPAP values in patients with PAH revealed that 62.5% of those values became inaccurate by overestimation, whereas in a group of patients with mild to moderate TR, the prevalence of inaccurate sPAP values reached only 37.5% [217]. Severe TR was also identified as the only independent predictor of sPAP overestimation by TTE [217]. In another study, although the sPAP values obtained in 138 PAH patients by both TTE and RHC were found to be highly correlated (r = 0.81, *p* < 0.01), in 58% of those patients, the TTE-derived sPAP values were inaccurate when compared to the sPAP values measured directly by RHC [213]. Strikingly, there was a high prevalence of highly relevant TR in the “inaccurate group” which, with 47.5%, was twice as high as that found in the “accurate group” [213]. In another study, a large group of patients with PAH where 86.7% had ≤moderate TR and only 6.7% had severe TR, although the TTE-derived sPAP showed only a moderate agreement with the sPAP measured by RHC, the TAPSE/sPAP ratio calculated in each patient via the TTE- and the RHC-derived sPAP value revealed a strong correlation [214]. Therefore, also important was the observation that despite the very low proportion of patients with relevant TR, the presence of severe TR increased the correlation between the sPAP values estimated by TEE and those measured by RHC [214]. This explains the stronger correlation between the TAPSE/sPAP values obtained by the two different methods when compared to the weaker correlation revealed by the TTE-derived and RHC-derived sPAP values, given that TTE often underestimates the sPAP in the absence of relevant TR [214] or overestimate sthe sPAP in the presence of severe TR [217,219]. Surprisingly, it appears that severe TR can strengthen the correlation between the TAPSE/sPAP indexes calculated in each person by using both the TTE-derived and the RHC-derived sPAP value, and the TAPSE/sPAP ratios derived solely from the TTE-derived parameters revealed an overall better prognostic performance than the TAPSE/sPAP based on the RHC-derived sPAP values [214]; this becomes explainable if the sPAP overestimation by TTE is also considered [217,219]. Thus, given that an overestimated sPAP can diminish or even abolish the TR-induced misleading increase in the TAPSE value [4,32,161,167], the overestimation of the TTE-derived TAPSE/sPAP ratio induced by the higher TAPSE can be counteracted by a concurrently overestimated sPAP value, whereas the RHC-derived TAPSE/sPAP ratio will remain overvalued by the TR-facilitated misleading increase in the TAPSE. In PH induced by high pre-capillary PVR, among the different potential surrogates for the non-invasive assessment of the RV-PA coupling, only the TAPSE/sPAP index has proved to be an independent predictor of Ees/arterial elastance and patients with TAPSE/sPAP values < 0.31 mm/mmHg revealed a significantly worse prognosis than those with higher TAPSE/sPAP values [140,208]. In patients with PAH, a recent study revealed that the evaluation of RV-PA coupling by using the TTE-derived TAPSE/sPAP ratio improved certain RV assessment risk scores, but with the exception of patients with the lowest and the most advanced stage of PAH [215]. These exceptions are understandable, given that ongoing RVPO initially leads to an adaptive concentric remodeling characterized by RV wall thickening with a reduction in RV wall tension associated with an increase in the myocardial contractility, in order to prevent the reduction in RV ejection into the PC (Figure 1), an adaptive response which is also reflected by the lack of TAPSE reduction [32]. With the aggravation of the RVPO, the increase in the PVR will be associated with a simultaneous increase in the sPAP and a decrease in the highly afterload-dependent TAPSE, thereby inducing a progressive reduction in the TAPSE/sPAP ratio [32]. However, in the most advanced stages of RVPO, the aggravation of maladaptive RV remodeling associated with a steadily declining myocardial contractility will induce a progressive reduction in the sPAP, even in the presence of a further increase in the PVR, whereas the RV dilation-induced TR aggravation can counteract the reduction in TAPSE [32,133]. All these can also explain the main reasons for the recently reported loss of the TAPSE/sPAP usefulness for risk assessment in patients with advanced stages of PAH [216].

The TAPSE/TRPG ratio appeared particularly useful for the evaluation of patients with estimated TRPG values ≤ 46 mmHg because of its high accuracy in screening for PH overall, but even more importantly because of its considerably higher accuracy than all other tested parameters (including, among others, the sPAP, TRPG and the RVOT-AT) in screening for pre-capillary PH in patients with PA wedge pressure [PAWP] ≤ 15 mmHg [135].

In patients with PAH, the TTE-derived RVFWS/sPAP was found to be more predictive of the clinical outcome than the TAPSE/sPAP and, whereas the RVFWS even appeared predictive alone, the TAPSE revealed no predictive value for the clinical outcome [220]. This is explainable, given that unlike TAPSE (a measure of TA displacement), the RVFWS (a measure of myocardial deformation) is unaffected by the motion of the entire heart or by that of certain myocardial regions [32]. As already mentioned, especially in PH with a severely dilated and dysfunctional RV, the rocking motion of the heart can induce highly misleading alterations of TAPSE not only before, but also after successful therapy [162,163]. Although TAPSE and RVFWS are both load-dependent parameters which reflect RV systolic changes in similar anatomic regions, the RVFWS, which quantifies only RV contraction-induced myocardial deformation by angle-independent measurements which are not affected by the myocardial displacement, is much more suited than TAPSE for inclusion into a combined parameter together with the ΔP_RV-RA_ [29]. In a more recent study involving patients with pre-capillary PAH, the ratio between the RVFWS and the RHC-derived sPAP value revealed high predictive values for both all-cause mortality and for outcome after combined HLTx, which were superior to those provided by TAPSE, FAC_RV_, RVFWS, as well as by TAPSE/sPAP and FAC_RV_/sPAP [221]. The RWFWS/sPAP ratio using the sPAP estimated by TTE also demonstrated the best accuracy for prediction of clinical endpoint, with a sensitivity of 92% and specificity of 82.5% for a cut-off value of 0.19 [221]. Patients with ratios > 0.19 had significantly better survival than those with ratios ≤ 0.19 [221].

In patients with acute HF, the impaired RV-PA coupling defined as a RVGLS/sPAP ratio below 0.32 appeared associated with a high risk of mortality across all HF phenotypes [222]. In a large study, including patients with severe functional TR in the presence of PH-LHD (estimated sPAP 56 mmHg [45–68 mmHg]), elevated RAP and less negative RVFWLS (≤−18%) were independent predictors of all-cause death [223]. The combination of high RAP and RVFWLS effectively stratified the all-cause death and the 600 days freedom from all-cause death was 47% higher in patients with RVFWLS > 18% with non-elevated RAP in comparison to those with RVFWLS ≤ 18% associated with elevated RAP.

The TTE-derived RV contraction pressure index [RVCPI], calculated by using the formula RVCPI = TAPSE × ΔP_RV-RA_, appeared strongly correlated with the RHC-derived RV stroke work [RVSW] and also revealed a high predictive value for the outcome for patients with advanced chronic HF [204,210]. In patients with acute decompensated chronic HF (LVEF < 30%) associated with PH-LHD (sPAP on average, 51 mmHg), a prospective study identified the presence of a low RVCPI as the best predictor of outcome, whereas neither the FAC_RV_ or TAPSE alone, nor the FAC_RV_/sPAP or TAPSE/sPAP ratios showed significantly predictive values [204]. These findings are quite logical because at a given PVR, any reduction in RV pump function will result in a reduction in the ΔP_RV-RA_ [29]. Thus, the TAPSE × ΔP_RV-RA_ can decrease, even if the RV contractile dysfunction-related TAPSE reductions are misleadingly attenuated or even nullified in patients with a severe TR [29]. Even when considered from a purely mathematical point of view, the simple multiplication of TAPSE with ΔP_RV-RA_ can (at least theoretically) lead to difficulties in the interpretation of certain results. Thus, in a patient with, for example, a normal TAPSE of 20 mm concomitantly with a normal ΔP_RV-RA_ value of 21 mmHg (e.g., 29 mmHg–8 mmHg), the calculated RVCPI value will attain 420 mm × mmHg, whereas in a patient with a low TAPSE of 14 mm concomitantly with a higher ΔP_RV-RA_ value reaching 30 mmHg (e.g., 46 mmHg–16 mmHg), the RVCPI will also reach 420 mm × mmHg. However, by contrast, the TAPSE/sPAP ratio calculated in the first and second patient (i.e., 0.69 vs. 0.30 mm/mmHg, respectively) would reveal a 56.5% lower TAPSE/sPAP value in the second patient, a result which would be more expected than the identical RVCPI values. Nevertheless, in more advanced stages of RVPO with impaired RV-PA coupling, the RVCPI alterations appeared to be increasingly more related to the severity grade of RV dysfunction [204]. Based on the available data, it appears reasonable to use both the TAPSE/sPAP and the RVCPI than to select only one of these two related but not identical parameters, given that the RVCPI also includes the RAP, whose diagnostic importance increases with the aggravation of RV dysfunction [204]. This would have the advantage that especially in the most advanced stage of RVPO when the TAPSE/sPAP ratio will become less reliable, the diagnostic value of the RVCPI will usually increase [204,215]. Thus, given the high complexity, the large variability and the multiple interconnections of the pathogenic factors involved in the RV responses triggered and thereafter continuously amplified by PO before, during and after the transition from adaptive to primarily maladaptive right-sided heart responses (structural and functional) until the development of RHF, it is not seldom that even a combined parameter cannot reliably estimate the severity of RV function and/or predict the outcome of certain patients with RVPO [32,224]. Nevertheless, in such cases, certain combined parameters like TAPSE/sPAP can significantly improve the predictive values of validated larger risk scores [225].

The TTE-derived afterload-corrected peak RVGLSR [Ac-RVGLSR], calculable with the formula Ac-RVGLSR = peak RVGLSR × ΔP_RV-RA_, is a well reproducible and easily measurable combined parameter for the evaluation of the RV contractile function, which reflects the linkage between the myocardial shortening velocity and RV load. It has the advantage over RVCPI that the GLSR is less dependent on RV afterload than the TAPSE [32,191]. In addition, as in the case of the RVFWS, the GLSR measurements are not affected by the movements of the RV (or parts of its walls) during the systole, which are not directly related to the myocardial contraction [6]. There are also indications that the peak systolic SR can be superior to the peak strain in the estimation of myocardial contractility because it appeared to be less affected by changes in cardiac load and structure [226]. Experimental studies revealed that, whereas the end-systolic myocardial strain is more sensitive to afterload than to intrinsic myocardial contractility, the peak systolic SR appeared primarily dependent on myocardial contractility, regardless of the loading conditions [227].

A simple additional approach to optimize the evaluation of RV responses to a steadily high and progressively increasing PO, triggered by an abnormal rise in the vascular resistance in the PC, can also be enabled by the RV load-adaptation index (LAI_RV_) [11,15,20,228,229,230,231,232,233,234,235,236,237]. This 2D-ECHO-derived composite index relies basically on the relationship between the RV dilation and its hemodynamic loading conditions, given that in patients with a similar SV index and resistance to blood flow in the PC, a less RV dilation indicates a better adaptation to the increased afterload [11]. Using for the LAI_RV_ calculation the easily measurable VTI_TR_ (i.e. velocity-time integral of the color-Doppler TR signal) as a surrogate for the hemodynamic load (i.e. for the pressure difference between the RV and RA [ΔP_RV-RA_]) and the end-diastolic RV area [A_ED_] as a surrogate for the less reliably measurable RV end-diastolic volume [RV_EDV_] for obtaining a better reproducible RV size-geometry index, allows the obtainment of a simple, dimensionless and good reproducible index. Therefore, the LAI_RV_, which reflects basically the relationship between the right-sided heart hemodynamic loading and the RV end-diastolic volume and geometry alterations (i.e. ΔP_RV-RA_ divided by the RV_EDV_/L_ED_ ratio, where L_ED_ is the long-axis lengths of the RV), is calculable by the formula:

LAI_RV_ = VTI_TR_ (cm) × L_ED_ (cm)/A_ED_ (cm^2^) (1)

**Figure 2:**
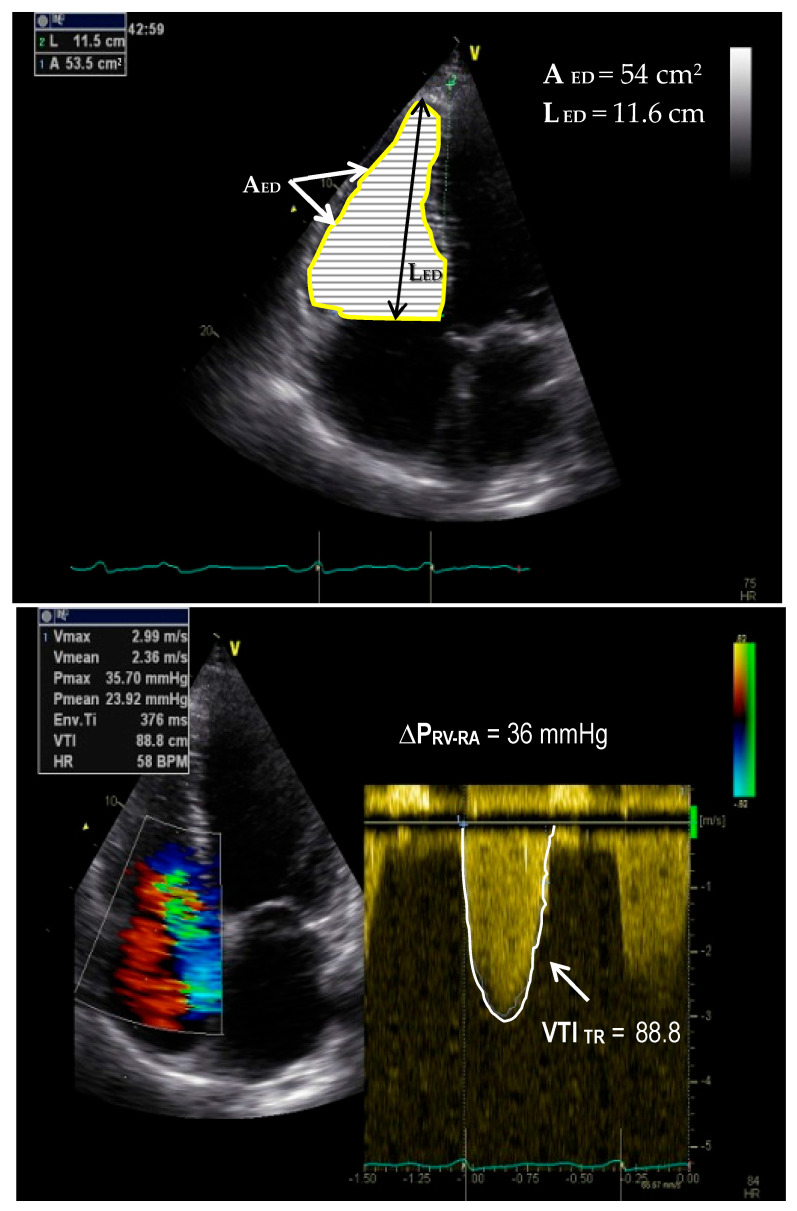
Calculation of the RV load-adaptation index from 2D-ECHO-derived measurements [11,15,20,32,60,149,226,227,228,229,230,234,235]. **Upper** image: Measurement of RV end-diastolic area and long-axis lengths (A_ED_ and L_ED_, respectively). **Lower** image: Measurement of the velocity–time integral (VTI _RV_) by Doppler-ECHO. Based on those measurements, the LAI_RV_ calculation results in a value of 19, which indicates a reduced RV adaptability to increased afterload, but with a still potential reversibility by reduction of its hemodynamic overloading.

Thus, the LAI_RV_ is, in fact, nothing else than a combination of conventional TTE-derived parameters which are usually measured during routine examinations. The use of the VTI_TR_ as a surrogate for the RV hemodynamic load instead of the ΔP_RV-RA_, which is in fact calculated from the mean velocity of the TR jet, allows not only the obtainment of a dimensionless index, but also has the advantage of including the duration of the regurgitant blood flow into the RA during the RV systole [11,20,152]. The inclusion of the end-diastolic and not the end-systolic RV area, as well as the end-diastolic long-axis length into the calculation formula, appeared more appropriate, especially in advanced RV overloading, since RV dilation is better quantifiable in the end-diastole (particularly in the presence of relevant TR, which leads to underestimation of the RV dilation in the end-systolic phase) [11,152]. A small RV area relative to the RV long-axis length (i.e., unaltered RV size and geometry) in persons with high VTI_TR_ (i.e., high RV systolic pressure without elevated RAP) generates higher LAI_RV_ values, which suggests a better adaptation to load (i.e., higher ability to rise the RV systolic pressure without either a significant RV dilation and/or a relevant rise in the RAP) and thereby also a probably good or at least adequate RV contractile function, with the ability of the RV to improve its pump function after reduction in the PO [11,62]. Conversely, a spherical RV dilation (i.e., a large RV area relative to its long-axis length), despite a rather low VTI_TR_ value, which suggests a greater increase in RAP than in RV systolic pressure, will generate a low LAI_RV_ which helps identify the existence of a poor adaptation to hemodynamic overloading (i.e., disproportionally severe RV dilation despite a relatively low RV pressure load, also indicating a reduced RV systolic function), thereby suggesting a reduced RV myocardial contractility. It was found that LAI_RV_ values < 15 indicate excessively low RV adaptability to load, making the RV unable to prevent RVF even in the presence of a normal afterload [11,29,32,62,148]. Figure 2 shows an example of 2D-TTE-derived measurements necessary for the calculation of the LAI_RV_ and also summarizes the essential principles for the interpretation of the TTE-derived measurements and the calculated index value.

Initially, the LAI_RV_ was introduced to improve preoperative decision-making in LVAD candidates with end-stage LVF associated with secondary RV dilation and dysfunction plus relevant TR, regarding the potential necessity for implantation of a biventricular MCS [11]. The usefulness of the LAI_RV_ was thereafter successfully tested in patients with severe PAH in order to improve the timing of DLTx listing [20]. Later, in a retrospective study on 194 adult continuous-flow LVAD recipients, the LAI_RV_ was found to be a strong predictor of clinical RHF not requiring additional RVAD implantation, revealing the strongest area under the receiver-operating curve among the ECHO- and RHC-derived measurements evaluated in the study, like the TAPSE/RVSP ratio (which revealed no predictive value), the RAP and even the RVFWS [15].

A prospective study involving 86 HFpEF patients divided into two groups (one with, the other without elevated PAWP) revealed that among the tested parameters used for RV evaluation (i.e., TAPSE, sPAP, sPAP/RVESA, TA systolic displacement velocity [TAS’], TAS’/sPAP, RVFWLS/sPAP and LAI_RV_), the resting/exercise RVFWLS/sPAP and the LAI_RV_ were found to be most strongly correlated with the PAWP (*p* < 0.001) [236]. By reflecting the RV-PA coupling, the LAI_RV_ appeared to be a robust predictor of adverse CV events in HFpEF associated with PAWP [236].

LAI_RV_ = VTI_TR_ (cm) × L_ED_ (cm)/A_ED_ (cm^2^) = 88.8 × 11.6/54 = 19 (2)

RV = right ventricle; 2D-ECHO = two-dimensional echocardiography; A_ED_ = end-diastolic RV area; L_ED_ = end-diastolic RV long-axis length; VTI_RV_ = Doppler-derived right ventricular velocity–time integral; LAI_RV_ = RV load-adaptation index. The preserved RV geometry (i.e., no increase in the A_ED_/L_ED_ ratio) despite its hemodynamic overloading (initially only pressure overloading, but over the course of time, also an additional volume-overloading due to the aggravation of the secondary tricuspid regurgitation), indicate the presence of a still preserved adaptability of the RV to face that pathological overloading. LAI_RV_ values ≤ 14 indicate an exhaustion of RV adaptability to pressure overloading. 

A comparative study involving two PAH groups, one with IPAH, the other with scleroderma-associated PAH [SSc-PAH], with similar baseline RVSP, CI, RV-A_ED_-index and RA area index values, but with higher sPAP baseline values in the IPAH group, revealed striking differences in the time course of the LAI_RV_ values after starting the pulmonary vasodilation therapy [PVT] [232]. Thus, although the baseline LAI_RV_ values were in the normal range in both groups and the average value in the SSc-PAH group was even nearly 30% higher than in the IPAH group, at one-year follow-up since starting the PVT, the LAI_RV_ increased in the IPAH group (with 36%) whereas it decreased in the SSc-PAH group with 15.6% [232]. Compared to SSc-PAH patients, those with IPAH revealed better RV reverse remodeling and 1-year outcomes [230]. Thus, whereas the event-free 1-year survival rate reached 95.5% in the IPAH group, in the SSc-PAH group, it was only 63.6%, despite the still normal LAI_RV_ average value in this group [232]. Among the investigated ECHO-derived parameters, only the LAI_RV_ and both the RV-A_ED_ and the RV-A_ES_ indexes revealed significant differences between their baseline values and their values at the last follow-up control [232]. Although none of the changes in the ECHO-derived RV metrics, which were observed after starting the PVT, were significantly associated with the primary endpoint, there were nevertheless significant differences (*p* < 0.01) between the two patient groups regarding the changes in the ΔLAI_RV_ (%) values (i.e., the difference between the baseline LAI_RV_ and LAI_RV_ values obtained at the last follow-up ECHO) [232]. These findings suggest the coexistence of a detrimental impact of intrinsic RV myocardial damages that are not caused by the high PVR can affect not only the outcome of patients with SSc-PAH, but also the diagnostic and prognostic value of certain metrics used for evaluations of the RV adaptability to PO. This is quite understandable given that in SSc, in addition to the afterload-induced secondary structural and functional RV alterations, there is also a high and usually rising prevalence of clinically overt cardiac myofilament dysfunction and pericardial damages induced by SSc-related inflammation and fibrosis, which can alter the innate responses of the RV to increased afterload [233,234]. Thus, the LAI_RV_ changes in opposite directions in the two patient groups deserves consideration because the LAI_RV_ may therefore become a useful tool for the early identification of PAH patients that are at a higher risk of additional intrinsic RV myocardial damage (like in SSc and other connective tissue diseases associated with PH and RV myocardial alterations [232,235]) with important prognostic impact.

Given the important impact of TR on RV size, geometry and function, as well as its impact on the prognosis of RVPO, these aspects also necessitate particular attention. Regarding this, a recent large multicenter study with the participation of 24 institutes from six European states (including 364 patients) revealed that the assessment of RV capacity to adjust for changes in loading conditions using the LAI_RV_ can better predict mortality in patients with ≥severe TR undergoing TV transcatheter edge-to-edge repair than other different classical parameters reflecting RV size and function like TAPSE, FAC_RV_ and TAPSE/sPAP [237]. The authors concluded that the LAI_RV_ appeared able to differentiate adapted and maladapted right ventricles and consequently to predict mortality in patients treated with T-TEER better than traditional metrics.

However, regarding the mentioned studies, it is important to take into consideration that the statistically more relevant results revealed by the LAI_RV_ were obtained within specific populations (e.g., HeartMate-3 LVAD candidates, SSc-PAH, HFpEF with elevated PAWP, TEER cohorts) and can therefore not be transposed to all patients with hemodynamic overloading-induced RV alterations. Overall, the studies discussed above revealed that even among the combined parameters, there are important differences in their strengths and weaknesses for the evaluation and prognostication of the highly different interactions between the RV and the pulmonary arterial circulation in patients with different types of pulmonary hypertension. Even the same combined parameter reveals often different diagnostic and prognostic values in the course of the disease. Thus, among the different RV-PA coupling/load-adaptation surrogates (including also the RVGLS/sPAP, RVFWS/sPAP and the LAI_RV_)_,_ none of them can currently be considered as a key parameter and their use should rather be a “complementary” than a “solely decisive” approach. In practice, the LAI_RV_ should be used alongside other measures (TAPSE/sPAP, RV strain-based ratios, eRVEF, risk scores) and not as a stand-alone decision tool. Also, before the adoption of a routine LAI_RV_ use in the TTE guidelines, prospective multicenter studies are mandatory. Table 3 provides a summary of the mainly used combined ECHO-derived parameters for initial evaluations of RV myocardial responses to hemodynamic overloading induced by pathologically high PVR values in the PC [11,15,20,64,152,191,199,200,201,202,203,204,205,206,207,208,209,210,211,212,213,214,215,216,217,220,221,222,223,224,225,226,227,230,232,233,234,235,236,237].

## 8. Prediction of RV Recovery After Abolition of Pressure Overloading

Prediction of RV reverse remodeling and functional improvement in case of a relevant reduction in its PO is particularly helpful in the decision-making before HTx and VAD implantation, as well as for the optimal timing of DLTx for patients with refractory end-stage PAH, before irreversible PO-induced RV alterations will lead to the need for a combined HLTx [13,14,15,17,18,19,20,21,65,83,89,119,121,152,228,231,238,239]. Basically, both invasive (i.e., RHC) and non-invasive examinations (i.e., first of all ECHO) are mandatory, especially before LVAD implantation in patients with end-stage HF associated with PH-LHD [14,15,17,65,90], before decision-making between DLTx and HLTx in patients with PAH associated with severe RHF [121,238,239], as well as for the assessment of RV recovery in patients with long-term LVAD support with a temporary RV mechanical support [240,241,242].

### 8.1. Non-Invasive Assessment of the RV in HF Patients Necessitating an LVAD Implantation

The vast majority of ECHO-derived variables identified as risk factors for PO-induced RVF alone appeared unable to predict not even fairly, neither the occurrence of RVF nor the freedom from RVF after LVAD implantation [14,152,194]. A major advantage of combined multiparametric approaches is the enabling of simultaneous assessments of anatomical and/or functional changes of the right-sided heart in relation with its hemodynamic loading conditions of the RV, which thereby allows combined interpretations of various right-sided heart alterations [32,152]. This in turn facilitates estimations of the RV contractile function and its adaptability to hemodynamic overloading, as well as the prediction of RV reverse remodeling and functional improvement after reduction in the RV afterload [32,152].

**Figure 3:**
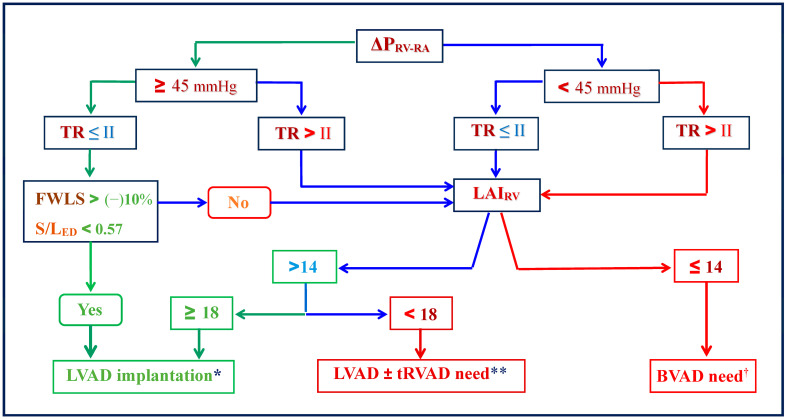
Facilitation of the decision-making for or against the necessity of an additional VAD support for the PH-LHD-induced secondary RHF in LVAD candidates with end-stage LV failure refractory to other medical or device therapy options, by considering the current impact of the RVPO on the RV size, geometry and the severity of TR [11,13,15,17,19,32,62,79,90,151,166,177,193,194,195,227]. PH = pulmonary hypertension; LHD = left heart disease; RHF = right heart failure; LV = left ventricle; LVAD = left ventricular assist device; RV = right ventricle; RVPO = RV pressure overloading; RA = right atrium; ΔP_RV-RA_ = difference between the RV and RA pressure during RV systole; TR = tricuspid regurgitation; FWLS = RV free wall longitudinal strain; S/L_ED_ = end-diastolic short/long-axis ratio; LAI_RV_ = RV load-adaptation index; RVAD = temporary right ventricular assist device; BVAD = biventricular assist device. * The unaltered RV geometry and size, despite the high hemodynamic overloading, indicates an optimal RV adaptation to load, able to improve RV function during the LVAD support. ** > moderate TR (i.e. TR > grade II) might be considered for surgical repair at the time of surgery. † LAI_RV_ values ≤ 14 indicate an irreversible exhaustion of RV adaptability to load, making the RV unable to prevent RV failure even in the presence of a normal afterload.

Given that end-stage HF involves both ventricles (even if its initial cause was a left-sided heart disease), with the increasing use of MCS, the necessity to create various parameter combinations and indices derived from measures obtained by non-invasive and/or invasive investigations in order to improve the decision-making between the necessity of an LVAD with or without the implantation of an additional VAD support for the most often secondary affected RV, became increasingly apparent [152]. Although LVADs provide better quality of life and also usually a longer survival probability than BVADs, it must be taken into account that RVF in LVAD recipients is often associated with renal, hepatic or multiorgan failure (even if the LVAD surgery is later followed by an additional RVAD implantation), which can often decisively contribute to the still relatively high mortality rates after LVAD implantation. [152,195,196] Thus, the identification of patients who require durable BVAD support, as well as those who only need a temporary RVAD [t-RVAD] in addition to the LVAD must occur preoperatively or at least not later than intraoperatively [11,62,79,85,178,228,231]. In the meantime, it became evident that combined ECHO parameters can be a cornerstone for the timely identification of patients in whom the RV is not anymore able to prevent RVF even in the presence of a normal afterload [12,13,14,15,17,19,26,62,82,84,90,167,194]. It is undoubtable that the assessment of RV responses to afterload changes must be the primary goal of the evaluation of patients for deciding between the use of an LVAD either alone or in connection with a temporary or a durable mechanical RV support [62,151,152]. Figure 3 provides a flowchart based on an overview of essential ECHO-derived parameters necessary for the facilitation and decision-making for or against the necessity of an additional temporary or durable VAD support for the RV in LVAD candidates with end-stage LVF and PH-LHD-induced secondary right-sided heart structural and/or functional abnormalities.

### 8.2. Assessment of RV Recovery in LVAD Recipients with a Temporary RVAD Support

In LVAD recipients who also necessitate a t-RVAD support due to the preoperative presence of advanced RVF secondary to a severe PH-LHD, the RVF is more likely to be reversible than a similarly severe LVF and the reversal of RV remodeling and its myocardial contractile dysfunction usually require only a short-term mechanical RV support [241]. In clinically stable patients with post-operative regression of RV dilation and no relevant TR and improvement of RV wall motion, the selection of patients for t-RVAD removal can be usually begin after 2–3 days of t-RVAD support [241]. The weaning is started with a gradual reduction in the RVAD flow (usually 0.5 L/day) to 2 L/min under ECHO guidance and monitoring of hemodynamic responses [11,241]. If the RHC and ECHO parameters remained stable during that moderate RVAD flow reduction, the next step will be an “off-pump trial” under an adequate anticoagulation comprising a short (5–10 min) reduction in the flow to 1 L/min followed by short interruptions of the RV support which are necessary for the actual assessment of RV recovery. If also under such conditions the ECHO parameters remain stable and RHC measurements remain normal, a stable RV recovery is very likely [11,241]. The major ECHO criteria for a possible removal of a t-RVAD are: a stable RV end-diastolic diameter at the RVOT < 35 mm, a RV short-/long-axis ratio < 0.6, a TAPS’ > 8 cm/s, a LAI_RV_ > 15, a TR ≤ 2 and no reduction in the VTI_RVOT_ during the off-pump trial, whereas at the same time, the RHC should reveal stable CVP and CI values of ≤12 mmHg and ≥2.4 L/min/m^2^, respectively [241].

### 8.3. Decision in Favor or Against the Necessity for HLTx in End-Stage PAH Concomitant with End-Stage RVF

For selected patients with therapy-resistant end-stage PAH or end-stage chronic thromboembolic PH [CTEPH] associated with end-stage RVF, DLTx or more rarely HLTx can provide the only therapeutic option for their survival [121,238,239]. Given that PO-induced RVF is the leading cause of mortality among patients with severe PAH and CTEPH, and the mortality rate of PAH patients with acute RHF can reach 40%, with the continuous prolongation of waiting times for Tx, early prediction of irreversible RVF in candidates for lung Tx is of crucial relevance for optimal timing of listing procedures, and therefore, the search for reliable prognostic predictors further remains a major goal [32,243,244]. In this regard, the prediction of RV reverse remodeling and a functional improvement after abolition of PO can be decisive not only for deciding between the necessity of a DLTx or a HLTx, but also for the most optimal timing of the DLTx in order to prevent the necessity of a HLTx in case of an excessive delay of the Tx listing procedure [20,32]. Because most inaccurate estimations of the ability of the RV to recover after DLTx (regardless of whether this was an under- or overestimation) can have life-threatening consequences on post-transplant results, in such critical cases, the routine use of integrated multiparametric invasive and non-invasive approaches for the evaluation of the right-sided heart can be of vital importance for patients [20]. Given that LAI_RV_ values < 15 indicate an excessively low RV adaptability to load, which makes the RV usually unable to avoid an RHF even after the a normalization of its afterload, this simple TTE-derived index deserves particular attention during the multiparametric pre-transplant evaluation of all DLTx candidates with severe PAH [11,29,32].

## 9. Conclusions and Perspectives

Although it is proven that PO-induced secondary RV dysfunction is one of the most important predictors of patient outcomes with PH-LHD PAH and CTPH, the complex and also temporally highly variable interactions between certain structural and functional changes in both the PC and in the hemodynamic overloaded right-sided heart, as well as between both ventricles, can often hamper the interpretation of certain measurement parameter changes and even relevantly alter their reliability. Moreover, the progressive aggravation of a secondary TR has an increasingly negative (often also misleading) effect on the diagnostic value of some of the most frequently used parameters for the estimation of right-sided heart alterations, secondary to the abnormally high hemodynamic overloading. It is therefore comprehensible that single parameters are not sufficiently reliable neither for the evaluation of RV dysfunction nor for predictions of its prognostic relevance across the whole spectrum of RVPO. By contrast, carefully selected integrated multiparametric approaches for the evaluation of RVPO had unequivocally proved to be much more reliable and are therefore also clearly more suitable for clinical use.

The continuous optimization of multiparametric approaches for the evaluations of PO-induced right-sided heart alterations has led to substantial improvements in the prediction of a preserved RV responsiveness to the abolition of its steady pressure overloading by a reversal of its maladaptive remodeling and a normalization of its pump function. Given the decisive importance of such a prediction, particularly for therapeutic decision-making in patients with end-stage PH-LHD, PAH and CTPH (especially in candidates for MCS implantation or thoracic organ transplantation), the multiparametric assessment of PO-induced right-sided heart alterations has become a mandatory requirement with a crucial impact on patient survival. Overall, the currently non-invasively and invasively obtainable parameters reflecting the individual structural, functional and hemodynamic characteristics of pulmonary circulation and both the right-sided and left-sided heart can usually provide sufficient information for an optimal evaluation. The interpretation of the major findings and the estimation of their prognostic impact is much more problematic because with the aggravation of the right-sided heart overloading, several parameters can lose their reliability and may become even misleading. However, among several directions and proposals for the future, the search in the direction of an all-driven multiparametric scoring system, or at least multiparametric scoring systems adapted to the current stage of RVPO, need to be taken into consideration.

For patients with suspected RVPO, the 2D-ECHO (including Doppler imaging, TDI and STE) is essential for initial diagnostic clarification. TTE is in this regard the first-line tool and it therefore remains indispensable for routine follow-up evaluations. After the initial diagnosis, followed by a close TTE monitoring, patients with relevant PH-LVH, and especially those with IPAH and indications of rapid progression of the disease, should be referred to a specialized center in order to start as early as possible with the most suitable therapy also adapted to the stage of the disease.

Based on the growing body of evidence for the important role of altered immune responses in the pathogenesis of both PH-LHD (especially in patients with myocarditis-related or idiopathic DCM) and PAH, during the last two decades many attempts were made to find clinically suitable immunotherapy approaches for the improvement of patient outcomes. In this regard, one of the most important results was the introduction of IA therapy for IDCM (which is sooner or later associated with PH-LHD) into the guidelines of the American Society of Apheresis where it achieved a “grade B” (i.e., strong) recommendation with the remark that “IA can be applied without reservation in most patients in most circumstances” [245]. Although IA has shown promising short-term hemodynamic and functional benefits in small IPAH series for the removal of AABs against α1-ARs and ETARs in AAB-positive patients with IPAH and also appeared well tolerated, it remains an investigational add-on therapy and is not included in current PAH guidelines. Nevertheless, given the compelling necessity to focus the therapeutic approaches much more on the pathobiological particularities of the IPAH and thereby also on the immunological factors involved in this still incurable disease, the use of IA and/or the intravenous injection of aptamers for AAB neutralization, as well as the potential use of heart-derived progenitor cells with relevant immunomodulatory and anti-inflammatory effects could be an important step forward, but further clinical studies are necessary before any generally applicable decision in this regard can be adopted.

Overall, for critical therapeutic decision-making processes, the assessment of RV adaptability to pressure overloading always remains of crucial importance, with increasing significance in the more advanced stages of the pressure overloading.

## Figures and Tables

**Table 1:** Cardiac autoantibody removal by immunoadsorption [92,93,94,97,99,100,102,103,104,105,106,107,108,109,110].

Methods	Basic Characterization
Plasmapheresis (PE) vs. Immunoadsorption (IA)	Cardiomyopathies are associated with a particular class of AAbs directed against G-protein-coupled receptors (GPCR-AAbs), which also incorporates the β1AR-AAbs with high prevalence in IDCM. PE and IA are extra-corporeal blood purification techniques used to remove pathogenic AAbs in autoimmune diseases, but IA is more selective, safer, and faster. IA removes specific immunoglobulins, preserving other plasma proteins. PE replaces all plasma, increasing the risk of adverse events like infection or thrombosis and is not suited for removal of cardiac AAbs.
Nonselective IA	This “unspecific” IA utilizes binding materials (ligands) to trap most IgG subclasses and IgM antibodies while returning the essential proteins, such as albumin and clotting factors, back to the patient.
Semi-selective IA	This semi-selective IA with respect to the removal of β1AR-AAbs targets immunoglobulins while sparing other important plasma proteins like albumin and coagulation factors. It removes a broad range of immunoglobulins (IgG1, 2, and 4, plus partly also IgG3), rather than just a single specific antibody, making it suitable for various autoantibody-mediated diseases like IDCM. It utilizes columns with high binding affinity to Ig subclasses (using protein A, protein G, or anti-human Ig antibodies). The commonly used systems are Immunosorba, Therasorb and Globaffin adsorber. The latter is designed to selectively remove immunoglobulins (specifically IgG) and immune complexes from patient plasma.
Selective IA	This epitope-specific IA targets only specific pathogenic molecules and can be performed with peptide columns (Coraffin, Affina) where the used peptides mimic the AAb-binding epitopes of the β1-AR, thereby allowing for selective elimination of β1-AAbs.

AAbs: autoantibodies; AR: adrenoreceptor; IDCM; idiopathic dilated cardiomyopathy.

**Table 2:** Overview of the main goals and the necessary ways to their achievement related to the right-sided heart assessment in persons with ongoing PO-induced RV dysfunction caused by high post-capillary and/or pre-capillary resistance in the PC [16,17,31,40,43,53,56,83,139,152].

Main Goals	Ways of Achieving the Clinical Objectives
Assessment of the current right-sided heart pathological changes and estimation of their prognostic impact	Evaluation and severity grading of the right-sided heart structural and/or functional pathological changes.
	Estimation of the current relative contribution of hemodynamic overloading to the right-sided heart pathological changes.
	Prediction of the most likely course of RV dysfunction as well as the long-term patient outcome.
Evaluation of the pressures in the right-sided heart and the resistance to blood flow in the pressures in the PC	Mandatory baseline RHC for the diagnosis of PH-LHD and PAH by measuring the PCWP, CO, systolic and diastolic PAP (with calculation of mPAP), RVP and RAP as well as by the calculation of TPG (i.e., mPAP–PAWP), the DPG (i.e., PAP–PAWP), and the PVR (i.e., mPAP–mean PAWP)/CO).
Optimization of therapeutic decision-making processes	Close monitoring of treatment results for individual adaptation of the therapy to the crucial goal of avoiding or at least delaying the RV decompensation.
Detection and estimation of RV adaptability impairment	Timely detection of RV adaptability exhaustion by hemodynamic overloading and RV transition to maladaptive remodeling with progression towards irreversible RVF.
Prediction of the RV ability to reverse its maladaptive remodeling and restore its function after abolition of hemodynamic overloading	Prediction of the reversibility of advanced PH-LHD-induced combined high pre- and post-capillary PVR before making a decision regarding the life-saving implantation of either an LVAD or a BVAD, or an LVAD plus a t-RVAD.
	Prediction of the reversibility of the PH-LHD-induced high pre-capillary PVR in candidates for heart transplantation.
	Decision-making between DLTx and HLTx in patients with PAH associated with severe RHF.

PO = pressure overloading; RV = right ventricle; PC = pulmonary circulation; RHC = right heart catheterization; RVF = RV failure; PH = pulmonary hypertension; LHD = left heart diseases; PAH = pulmonary arterial hypertension; PAWP = pulmonary arterial wedge pressure; CO = cardiac output; PAP = pulmonary arterial pressure; mPAP = mean PAP; PVR = pulmonary vascular resistance; RAP = right atrial pressure; TPG = transpulmonary pressure gradient; DPG = diastolic pressure gradient; LVAD = left ventricular assist device; BVAD = biventricular assist device; t-RVAD = temporary right ventricular assist device; DLTx = double lung transplantation; HLTx = combined heart and lung transplantation; RHF = right heart failure. Whereas initially the adaptive right-sided heart responses are associated with increased myocardial contractile function, in the course of the disease, the progressive reduction in RV contractile function will become increasingly more relevant for patient outcomes.

**Table 3:** Overview of the major ECHO-derived combined parameters with and without additionally included RHC-derived measurements for the evaluation of RV myocardial responses to hemodynamic overloading initially induced by pathologically high pulmonary vascular resistance [11,15,20,64,152,191,199,200,201,202,203,204,205,206,207,208,209,210,211,212,213,214,215,216,217,220,221,222,223,224,225,226,227,230,232,233,234,235,236,237].

Parameters	Particularities and Advantages	Limits
TAPSE/sPAP (i.e., relationship between TA peak systolic excursion and pulmonary arterial pressure)	- Enables estimations of RV systolic function in relation with its current afterload by assessing the relationship between the longitudinal displacement of the TV_Ann_ and the necessary pressure to overcome the resistance to blood flow in the pulmonary vessels. - Correlates with the invasively estimated RV systolic elastance/arterial elastance.	- Its reliability is limited by TR which induces misleading increases in TAPSE, corresponding to the increasing regurgitant volume during the course of RV hemodynamic overloading. - Necessitates additional estimation of the RAP for the ECHO-based calculation of the sPAP. - Both limitations can impair the reliability of TAPSE/sPAP ratio for mortality prediction in patients with HF induced by primary impaired LV function.
RV contraction pressure index (RVCPI) RVCPI = TAPSE × ΔP_RV-RA_	- ECHO-derived RVCPI showed a significant correlation with the RHC-derived SWRV and revealed a high degree of predictability for a depressed SWRV. - In a study where neither TAPSE/sPAP nor FAC_RV_/sPAP revealed significant predictive values, a reduced RVCPI was the best predictor of outcome. - In more advanced RVPO with impaired RV-PA coupling, the RVCPI alterations are increasingly more relevantly related to the severity grade of RV dysfunction.	- Its reliability can also be impaired by severe TR which induces potentially misleading increases in the TAPSE values. - A simultaneous PO-induced reduction in TAPSE and increase in ΔP_RV-RA_ in similar proportions will not affect the calculated RVCPI values.
RHC-derived RV ejection efficiency (RVEe): RVEe = TAPSE/PVRi ECHO-derived RVEe: RVEe = TAPSE/PVR PVR = TR_peak velocity_/VTI_RVOT_	- In clinically stable pediatric patients, both the RHC-derived and the ECHO-derived RVEe are inversely related to the NYHA FC. - A significant (*p* < 0.001) negative correlation between the TAPSE and both RHC-derived PVRi and sPAP was also found.	- The clinical usefulness of the RVEe calculated solely from ECHO-derived measurements is currently not established. - In addition to the potential misleading impact of severe TR on the TAPSE, the frequent impairment of VTI_RVOT_ measurements by the high angle dependency of the used technique should also be considered.
RV afterload-corrected peak global longitudinal strain rate (Ac-GLSR): Ac-GLSR = peak GLSR × ΔP_RV-RA_	- By including the STE-derived myocardial deformation velocity (i.e., GLSR), this parameter has the advantage of reflecting only the myocardial responses induced directly by myocardial contraction and is not affected by the movement of the entire heart or by passive displacement of certain parts of the RV wall (particularly the IVS) compared to TAPSE and TAPS’.	- Although the combination of the GLSR with ΔP_RV-RA_ results in a less afterload-dependent parameter (i.e., Ac-GLSR) in comparison to RVCPI, it should be taken into consideration that the GLSR is not afterload-independent and its combination with the load-dependent ΔP_RV-RA_ reduces its value for potential estimations of changes in myocardial contractility.
RV stroke work index (RVSWI): RHC-derived RVSW: RVSWI = SVi × (mPAP − mRAP) × 0.0136 ECHO-derived RVSW: RVSW = 4 × [TRj peak velocity]^2^ × [PVA × VTI]	- By incorporating the SV, PAP and RAP, the RVSWI reflects the total RV work-load and can therefore be useful for prediction of patient outcome with PAH. - The ECHO-derived RVSWI revealed a significant (*p* < 0.001) correlation as well as a high concordance of its absolute values with the RHC-derived RVSWI.	- ECHO-derived RVSV calculations using the SV measured at the RVOT at the level of PV annulus are usually less reliable than the ECHO-derived calculations of the LVSV.
RV load-adaptation index (LAI_RV_) LAI_RV_ = [VTI_TR_ (cm) × L_ED_ (cm)] /A_ED_ (cm^2^)	- This simple and reproducible ECHO-derived dimensionless index relies on the relationship between RV dilation and its hemodynamic loading conditions, given that in patients with similar SVi and resistance to blood flow in the PC, a lesser dilation of a pressure-overloaded RV indicates a better adaptation to higher afterload. Together with the other parameters, this index is can be very useful. - An important advantage is its increasingly diagnostic and predictive value with the aggravation of the PO-induced RV alterations (including the TR severity) for both the patient outcome and the preserved ability of the RV for reverse remodeling and functional improvement after abolition of the excessive PO.	- Given that its predictive value depends mainly on the extent of the PO-induced RV maladaptive remodeling and the severity of the secondary TR, the LAI_RV_ is less suitable as a diagnostic and prognostic tool during the early phases of the disease. - A quite understandable limitation of the LAI_RV_ is its somewhat less reliable predictive value of the RV ability for reverse remodeling and functional improvement after abolition of its pressure overloading in certain types of PAH (e.g., SSc-PAH) associated with altered innate RV responses to high afterload.

## Data Availability

No new data were created or analyzed in this study.

## Abbreviations

AABs: autoantibodies; Ac-RVGLSR: afterload-corrected peak RVGLSR; A_ED_ and A_ES_: end-diastolic and end-systolic area, respectively; AR: adrenoreceptor; AT1-AAB: angiotensin II type 1 receptor AAB; BVAD: biventricular assist device; CDCs: cardiosphere-derived cells; CI: cardiac index; CMR: cardiac magnetic resonance; CO: cardiac output; Cpc-PH: combined pre- and post-capillary PH; CT: computed tomography; DCM: dilated cardiomyopathy; DLTx: double lung transplantation; ECHO: echocardiography; Ees: end-systolic elastance; EF: ejection fraction; EI: eccentricity index; eRVEF: effective RVEF; ETARs: endothelin-1A receptors; FAABs: functional AABs; FAC_RV_: RV fractional area-change; fRVEF: forward RVEF; fSV: forward SV; HFpEF and HFrEF: heart failure with preserved EF and reduced EF, respectively; HTx: heart transplantation; HLTx: combined heart–lung Tx; IA: immunoadsorption; IPAH: idiopathic PAH; Ipc-PH: isolated post-capillary PH; IVA: isovolumic acceleration; IVCV: isovolumic contraction peak velocity; IVS: interventricular septum; LAI_RV_: RV load-adaptation index; LHF: left-sided HF; LTx: lung transplantation; LV: left ventricle; LVAD: LV assist device; LVF: LV failure; MCS: mechanical circulatory support; ΔP_RV-RA_: difference between the RVSP and RAP; mPAP: mean pulmonary arterial pressure; M/V: mass to volume ratio; MRI: magnetic resonance imaging; PA: pulmonary artery; PAC: pulmonary arterial circulation; PAH: pulmonary arterial hypertension; PAWP: PA wedge pressure; PC: pulmonary circulation; PEA: pulmonary endarterectomy; PET: positron emission tomography; PH-LHDs: pulmonary hypertension associated with left-sided heart diseases; PV: pulmonary valve; PVR: pulmonary vascular resistance; RA: right atrium; RAP: right arterial pressure; RegVol: regurgitant volume; RHC: right heart catheterization; RHF: right heart failure; RVCPI: RV contraction pressure index; RVEF: RV ejection fraction; RV_EDV_: end-diastolic volume; RVEDP: RV end-diastolic pressure; RV: right ventricle; RVF: RV failure; RVFW: RV free wall; RVFWS and RVFWSR: RVFW peak systolic longitudinal S and SR, respectively; RVGLS and RVGLSR: global RV longitudinal peak systolic S and SR respectively; RVH: RV hypertrophy; RVOT-AT: RV outflow tract acceleration time; RVPO: RV pressure overloading; RVSP: RV systolic pressure; RVSW: RV stroke work; sPAP: systolic pulmonary arterial pressure; STE: speckle-tracking ECHO; S and SR: STE-derived strain and strain rate, respectively; SV: stroke volume; TA: tricuspid annulus; TAPSE: lateral TA peak systolic excursion; TAPS’: lateral TA peak systolic motion velocity; TDI: tissue Doppler imaging; TEE: transesophageal ECHO; TPG: transpulmonary pressure gradient; TR: tricuspid regurgitation; TRPG: TR pressure gradient; t-RVAD: temporary RVAD; TRVmax: TR jet maximal velocity; TTE: trans-thoracic ECHO; TV: tricuspid valve; VCTR: TR vena contracta; VTI_TR_: TR velocity–time integral.
